# Porous Organic Cages

**DOI:** 10.1021/acs.chemrev.2c00667

**Published:** 2023-04-06

**Authors:** Xinchun Yang, Zakir Ullah, J. Fraser Stoddart, Cafer T. Yavuz

**Affiliations:** † Faculty of Materials Science and Energy Engineering/Institute of Technology for Carbon Neutrality, Shenzhen Institute of Advanced Technology (SIAT), Chinese Academy of Sciences (CAS), Shenzhen 518055, China; ‡ Shenzhen Key Laboratory of Energy Materials for Carbon Neutrality, Shenzhen Institute of Advanced Technology (SIAT), Chinese Academy of Sciences (CAS), Shenzhen 518055, China; § Convergence Research Center for Insect Vectors, Division of Life Sciences, College of Life Sciences and Bioengineering, Incheon National University, Incheon 22012, South Korea; ε Department of Chemistry, Northwestern University, 2145 Sheridan Road, Evanston, Illinois 60208, United States; τ School of Chemistry, University of New South Wales, Sydney, New South Wales 2052, Australia; ζ Stoddart Institute of Molecular Science, Department of Chemistry, Zhejiang University, Hangzhou 310027, China; ∥ ZJU-Hangzhou Global Scientific and Technological Innovation Center, Hangzhou 311215, China; φ Oxide & Organic Nanomaterials for Energy & Environment Laboratory, Physical Science & Engineering (PSE), King Abdullah University of Science and Technology (KAUST), 4700 KAUST, Thuwal 23955, Saudi Arabia; η Advanced Membranes & Porous Materials Center, PSE, KAUST, 4700 KAUST, Thuwal 23955, Saudi Arabia; Ψ KAUST Catalysis Center, PSE, KAUST, 4700 KAUST, Thuwal 23955, Saudi Arabia

## Abstract

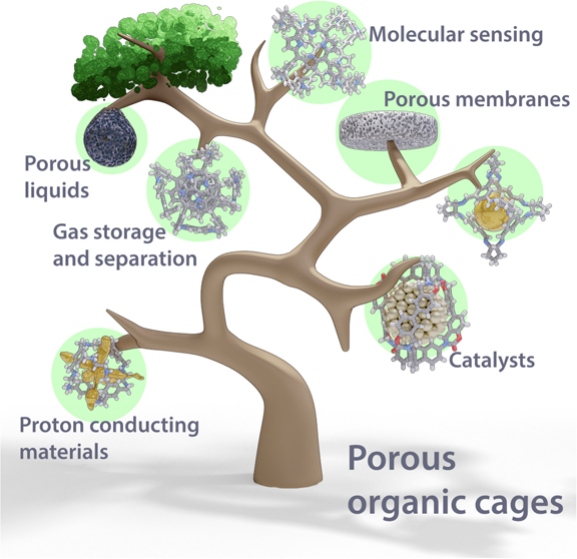

Porous organic cages (POCs) are a relatively new class of low-density crystalline materials that have emerged as a versatile platform for investigating molecular recognition, gas storage and separation, and proton conduction, with potential applications in the fields of porous liquids, highly permeable membranes, heterogeneous catalysis, and microreactors. In common with highly extended porous structures, such as metal–organic frameworks (MOFs), covalent organic frameworks (COFs), and porous organic polymers (POPs), POCs possess all of the advantages of highly specific surface areas, porosities, open pore channels, and tunable structures. In addition, they have discrete molecular structures and exhibit good to excellent solubilities in common solvents, enabling their solution dispersibility and processability—properties that are not readily available in the case of the well-established, insoluble, extended porous frameworks. Here, we present a critical review summarizing in detail recent progress and breakthroughs—especially during the past five years—of all the POCs while taking a close look at their strategic design, precise synthesis, including both irreversible bond-forming chemistry and dynamic covalent chemistry, advanced characterization, and diverse applications. We highlight representative POC examples in an attempt to gain some understanding of their structure–function relationships. We also discuss future challenges and opportunities in the design, synthesis, characterization, and application of POCs. We anticipate that this review will be useful to researchers working in this field when it comes to designing and developing new POCs with desired functions.

## 1 Introduction

Natural porous materials, such as diatoms, charcoal, cotton, feathers and sea-sponges, have been used widely for cooling, filtration, cleaning, and purification of water for many millenia.^1,2^ It is clear that their discrete porous structures endow them with the desired features of low density, large surface area, high thermal insulation, and excellent permeability. It comes, therefore, as no surprise that the evolving needs of human civilizations have led to the controlled fabrication of porous materials. For instance, porous zeolites have been designed as both adsorbents and catalysts for ion-exchange, molecular separations, and petrochemical cracking,^3^ while urethane foams have been utilized as raw materials for seats, mattresses, and insulation boards.^4^

More recently, designed porous materials, such as metal–organic frameworks^5^ (MOFs), covalent organic frameworks^6^ (COFs), and porous organic polymers^7^ (POPs) with open, permanent, and interconnected pores have been introduced, enabling access to previously unavailable architectures and ground-breaking applications, including but not limited to sensing, shape- and size-selective gas adsorption and separation, energy storage and conversion, water purification, drug delivery, and heterogeneous catalysis.^8−13^ Additionally, as templates or precursors, they have enabled production of a vast array of chemical compounds that have aided and abetted the development of conventional materials, such as carbon allotropes,^14^ metal oxides,^15^ metal nanoparticles^16^ and supported single-metal-atom catalysts.^17^ The remarkable feature of these structurally diverse porous materials is that they are readily extendable into two- (2D) or three-dimensional (3D) frameworks, in which the molecular building blocks are interlinked through strong dative or covalent bonds.^18−20^ These developments raise the question—are there any porous materials made up of small discrete molecules with open pores or cavities that can be permeable to gases or liquids? It is prudent to infer that porous molecular materials ought to be akin to, or even exceed, extended porous frameworks with respect to their porosity and potential applications for the simple reason that they can combine the merits of both classes of materials.

Porous organic cages (POCs) have emerged onto the scene as a new class of low-density crystalline materials composed of stable and permanent voids inside their rigid molecular structures that are equipped with windows to access external environments. Not unexpectedly, they have attracted a lot of attention during the past decade.^21,22^ They are constructed for the most part of covalent bonds, such as those between carbon–carbon or carbon–heteroatoms—e.g., imines, boronic esters, and amides—commonly found in organic molecules. In contrast to extended porous frameworks, POCs are synthesized and characterized in the first instance as molecular species, and then assembled into materials in the solid state, attaining almost all the advantages of emergent porous materials,^8^ e.g., high surface areas and pore volumes as well as open and tunable pores. Discrete molecular structures confer excellent solubilities upon their compounds in ordinary solvents along with high solution dispersibility and processability,^23^ rare features that are unattainable by relying on the mainstream, insoluble extended porous frameworks, such as MOFs, COFs and POPs. All these desirable features come with a hefty price, however, since the making of POCs is synthetically challenging. When compared with extended porous frameworks, the discrete POC molecules prefer to form thermodynamically stable products, often leading to nonporous or closely packed reticulated structures. As more researchers have taken up this challenge, the scope of POCs has broadened immensely in recent years, both in terms of their synthesis and applications. POCs have grown beyond the sole purpose of encapsulating guests and differ distinctively from cavitands and cryptands, even although other researchers^24^ continue to define them as such. In this review, we assess the recent research progress in the development of POCs from the standpoint of their strategic design, precise synthesis, advanced characterization and particular applications. Some representative POCs are selected as powerful examples in order to realize an in-depth understanding of the advantages and disadvantages of contemporary synthetic methods, the relationships between their microstructures and key functions, as well as their distinctive characteristics that sets them apart from extended porous frameworks. We discuss the challenges and opportunities of POCs in considerable detail. We anticipate that this review will guide researchers toward the rational design and development of novel POCs, as well as their hybrid/derived materials with potential for industrial applications in the near future.

## 2 Strategic Design of Porous Organic Cages

It is commonly understood that nonporous molecules can be packed into porous materials as a result of noncovalent bonding interactions, forming extrinsic porosity by nonideal assemblies.^25−27^ This situation is often attributed to the inefficient packing of inelegant molecular structures, for instance, the star-,^28^ propeller-^29,30^ or paddlewheel-like molecules,^31^ and hydrogen bonded ones,^32^ with directional intermolecular interactions. Researchers have designed and constructed a large number of porous molecular materials with extrinsic porosity, including Dianin’s compounds,^33,34^ Noria,^35^ tris(*o*-phenylenedioxy)cyclophosphazene,^36^ phthalocyanines,^37^ and hydrogen-bonded organic frameworks.^38^ Porous coordination cages, also known as metal–organic polyhedrons (MOPs) with intrinsic pores,^39,40^ are a subclass of supramolecular cages that can be constructed by a modular approach from metal cations and organic linkers. On account of the inner cavities and open windows, porous coordination cages have attracted significant attention in molecular recognition,^41,42^ gas adsorption,^43^ catalysis,^44^ and biomedicine.^45^ By virtue of the inherent cavities and pores, and complete organic backbones, the POCs are clearly distinguishable from extrinsically porous molecules and porous coordination cages. Note that this review excludes the porous molecular materials of extrinsic porosity and porous coordination cages.

**Figure 1:**
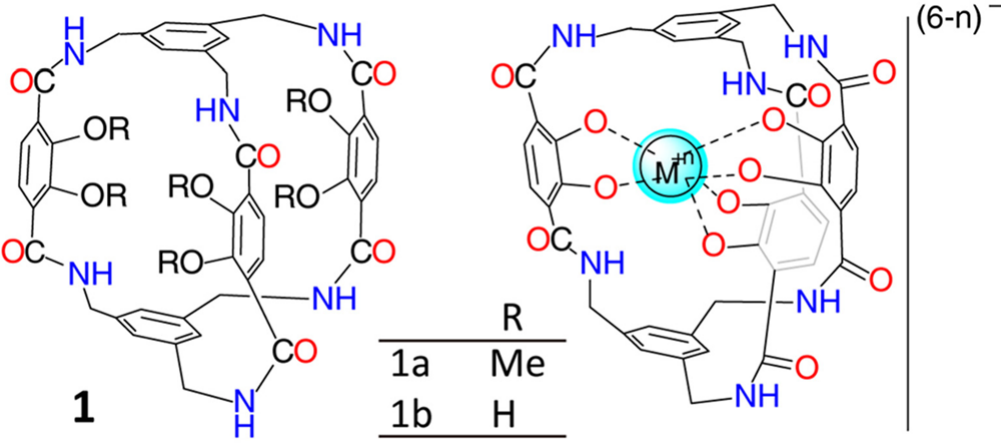
Construction of shape-persistent cage **1a** as a good siderophore candidate. Reproduced with permission from ref (47). Copyright 1984 Wiley-VCH.

The first examples of POCs were described in the literature in the 1970s. For instance, a hydrocarbon cage in a low yield of 1.7% with a complete C–C backbone was reported^46^ for the first time in 1977. Later on, a shape-persistent cage **1a** (Figure 1) with a mixed backbone of C–C and C–N bonds was reported^47^ as a good siderophore candidate in 1984. No credible attention, however, was given to their solid-state porosity. One reason could have been the lack of appropriate instrumentation in synthetic materials laboratories at that time to evaluate their crystal structures and porosities. Also, another reason could have been the targeting of the research toward the capture of substrates in solutions. By the same token, the recent rush on the rapid development of POCs benefits from the popularization of advanced gas adsorption and X-ray diffraction techniques that have become readily available in our times. Since the pioneering works on the synthesis of POCs for the adsorption of volatile compounds and gases were published by Atwood,^48^ Mastalerz,^49^ and Cooper^50^ in 2008 and 2009, a vast number of POCs have been designed and synthesized using established protocols.^21−26^

One fundamental advantage of POCs is that they are discrete molecular structures with internal voids and open windows. Their backbones are designed from C–C or C–heteroatom bonds, which are commonly present in organic molecules. The following questions, therefore, should be kept in mind when designing and synthesizing new POCs: (a) How do we select the organic linkers for a hypothetical cage? (b) What is the most favorable bond-forming chemistry and reaction conditions, e.g., precursor concentration, reaction solvent and the order or speed of precursor addition? (c) Will the cages maintain their porosity or collapse, when the solvent molecules are removed from their cavities? (d) What would be their crystal packing arrangements in the solid state? and (e) What physicochemical properties of POCs are to be expected? In short, a whole host of factors need to be contemplated so as to pursue the best balance between their structural stability and useful properties. In this regard, computational methods have brought a lot of promise to the design of POCs.^51^

### 2.1 Intuitive Design

**Figure 2:**
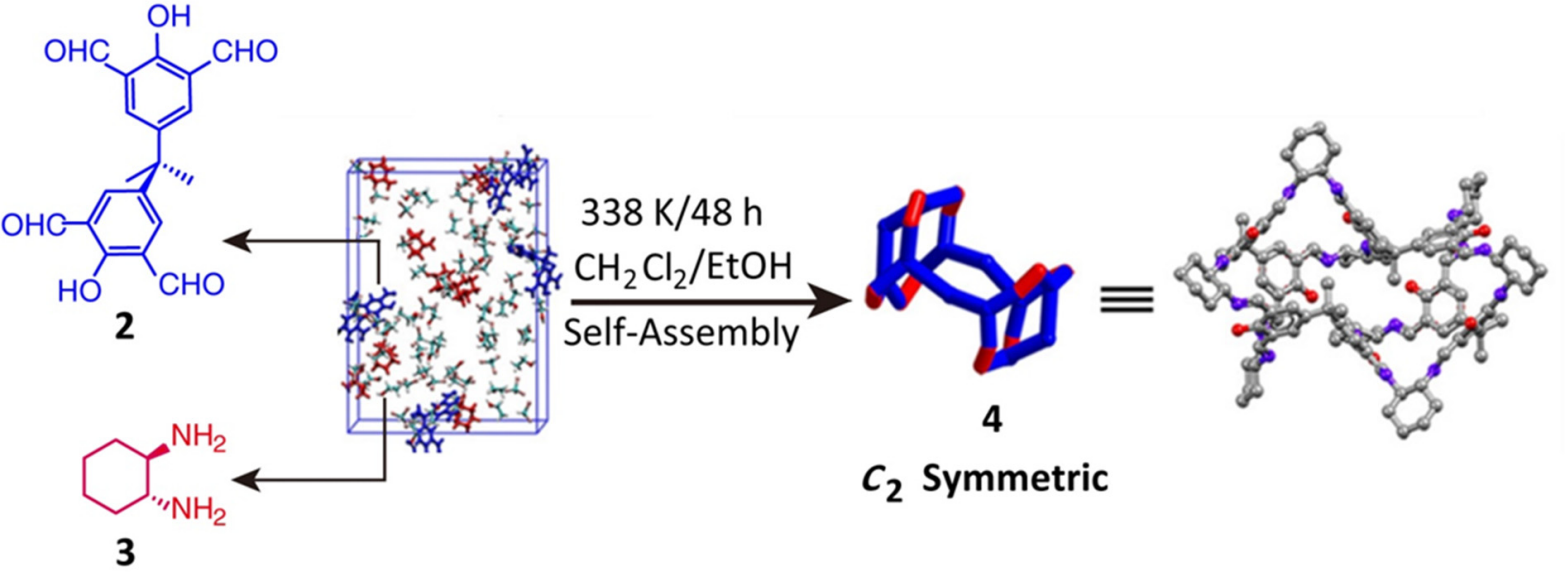
Synthesis of a C_2_-symmetry [4 + 8] organic molecular cage **4** by multidentate organic linkers. Reproduced with permission from ref (52). Copyright 2020 Wiley-VCH.

Choosing the appropriate organic linkers is the key to the design and development of POCs. In principle, the organic linkers should have two or more linking sites. Until now, most reported POCs have been constructed from two- or three-way organic linkers.^21−23^ Multidirectional organic linkers with more than 3-fold connectivity, however, are also known. An elegant example of a cage based^52^ on a multidirectional organic linker is the unusually low-symmetry [4 + 8] molecular cage, **4**, which was self-assembled (Figure 2) from the four-dentate aldehyde **2** and the two-dentate amine **3** in a mixed solution of dichloromethane and ethanol.

In order to form a cage, the preferred geometry of the organic linkers is of particular importance. Conventionally, wider bond angles in organic linkers lead to the formation of larger molecular cages, while the narrower bond angles in organic linkers afford comparatively smaller molecular cages.^53^ It should be noted that a slight change in the bond angle of organic linkers can have a dramatic effect^54,55^ on the crystallization of POCs. In addition, long-chain organic linkers tend to produce large molecular cages with high surface areas and porosity. This outcome, however, comes at a price—the resulting molecular cages are often prone to collapse^49^ after the removal of the solvent molecules trapped inside their cavities. The reason for the collapse can be attributed to the accumulated flexibility of many chemical bonds that constitute the cage framework. As a result, we often witness small, rigid molecular cages reported in the literature^56^ rather than larger ones since the former can be easily constructed by using π-bond-restricted, soluble organic linkers.

**Figure 3:**
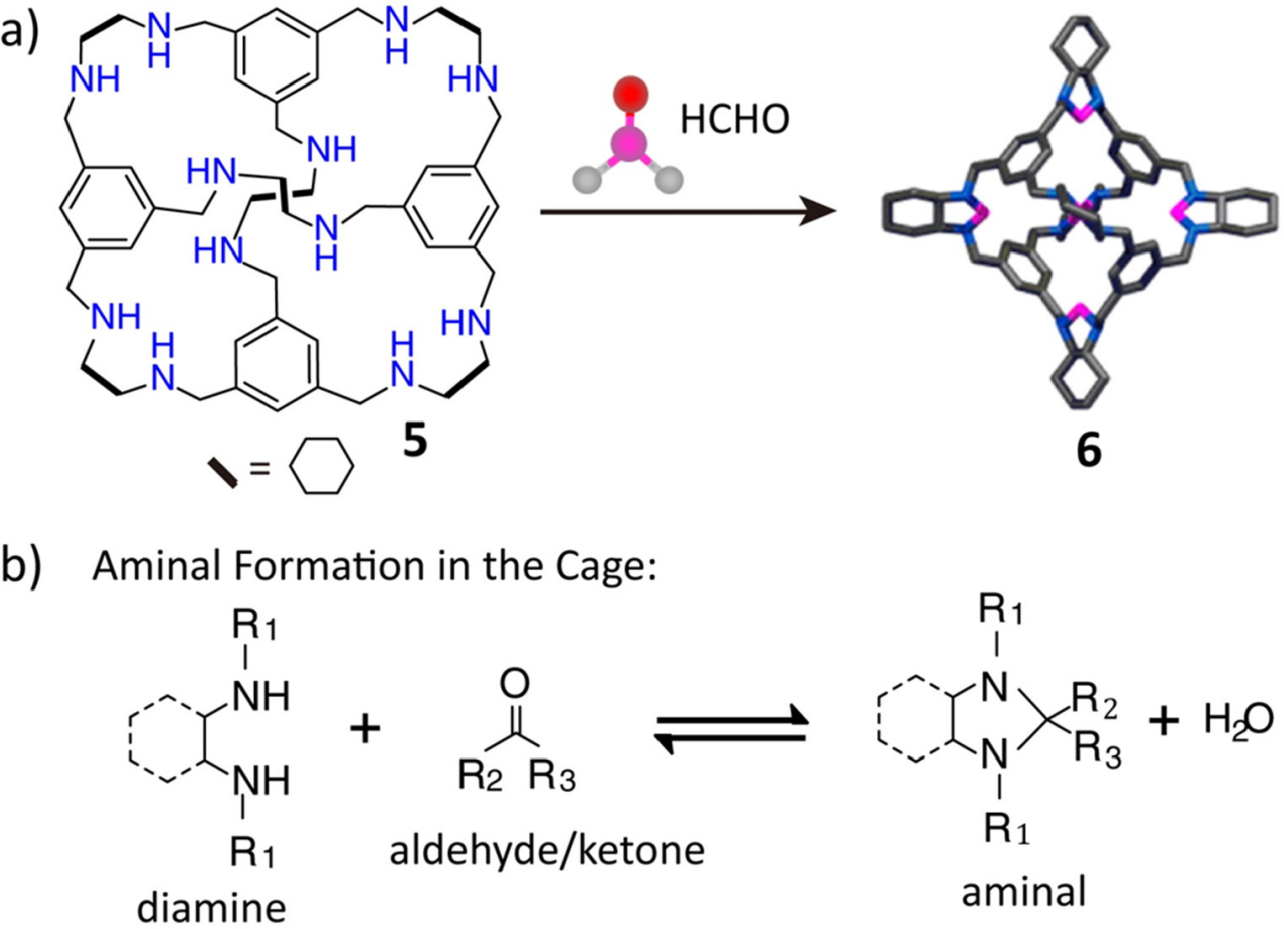
Synthesis of a “tied” porous cage **6**. Reproduced with permission from ref (64). Copyright 2014 American Chemical Society.

Once the organic linkers have been chosen, suitable bond-forming chemistry needs to be identified in order to construct the target POCs. Generally, the bond-forming routes to follow toward POCs can be classified into the following two categories: (i) irreversible linking chemistry and (ii) dynamic covalent chemistry. Irreversible linking chemistry often involves the formation of strong bonds, including C–C bonds,^57,58^ amide bonds,^59^ and bonds resulting from nucleophilic aromatic substitution.^60^ The advantage of the approach using the irreversible linking chemistry is that it can lead to the formation of highly robust POCs. The resulting cages, however, are often obtained in quite low yields, and therefore, need a complicated purification process before being ready for use. Dynamic covalent chemistry (DCC) involves the formation of weak, reversible chemical bonds, e.g. imine condensation,^49,50,52,53^ boronic ester condensation,^61^ and alkyne metathesis.^54,62,63^ The advantage of DCC is that it can afford high-yielding, high-purity POCs. On the flipside, the resulting cages have quite low chemical and thermal stability. Thus, in terms of the selection of bond-forming chemistry, a trade-off between structural stability, target yield, and desirable properties is usually unavoidable. An elegant solution for this trade-off^64^ is the use of a hybrid route (Figure 3), where prefabrication of the organic molecular cage **5** through reversible imine condensation is followed by the subsequent locking using irreversible linking chemistry, depicted in Figure 3b, to synthesize a robust POC **6**. The resulting cages were obtained in high yields (67%) and possessed remarkable stability in the pH range of 2.0–12.0, where most organic molecular cages collapse to give nonporous residues.

Irrespective of the nature of the linking chemistry, the reaction conditions are also crucial to the synthesis of POCs. The important parameters are the temperature, solvent, concentration of organic linkers, atmosphere, catalyst, and even the order and speed of the addition of organic linker. Any error in selecting the reaction parameters will lead to the formation of COFs, POPs, or other undesirable products, instead of the targeted molecular cages. In addition, the selection of reaction parameters has to take into account the intrinsic properties, such as their reactivity and solubility, of the organic linkers. Although it is commonly accepted that POCs are formed in organic solvents under dilute concentrations and mild temperatures with occasionally prolonged reaction times,^65^ other reaction conditions are also possible. For example, by functionalizing the organic linkers with solubility-promoting alkyl chains,^56^ a new range of reaction solvents is opened up, although the molecular cages may have low crystallinity and porosity. Another approach involves taking advantage of multivalency and ligand preorganization, e.g., the molecular cages have been self-assembled in water^66^ through the condensation of hexaformyl and bisamine derivatives, even although imines are not all that stable in water. In other cases, catalysts, such as metal salts and acids, are required to improve the kinetics of formation, crystallization, and product yield of POCs. The key issue, however, is that one must control strictly the type and amount of the added catalysts,^67^ since deviations from the ideal proportions will lead to the formation of undesirable products. Alternatively, rapid-action catalysts can minimize the adverse effects of reaction conditions. For example, poly(ionic liquid)s functionalized with carbene sites have been found^68^ to accelerate the crystallization rate of imine-linked POCs by a factor of at least 10-fold, compared to the more traditional trifluoroacetic acid (TFA) catalyst.

### 2.2 Computational Design

The design and synthesis of organic compounds, organometallics, and POCs require a lot of time, money, and raw materials, mainly because of the need to optimize chemical pathways. In order to avoid the experimental optimization cost from repetitive trial-and-error, computational tools^51^ can be employed to predict the best approaches to chemical reactions before starting lengthy experimental work. Theoretical and computational hypotheses for the prediction of POCs is mainly based on the following two key approaches:^51^ (i) classical force field methods and (ii) electronic structure calculations. Each theoretical methodology has its pros and cons. Typically in a classical mechanics approach,^69^ the force field expresses the energy of a system as the sum of intermolecular and intramolecular energies, while neglecting the electronic degrees of freedom. Modern quantum mechanical methods, on the other hand, can simulate the electronic structure,^51^ providing extraordinary insight into some of the characteristics, such as optical, electronic, and magnetic properties, that cannot be gleaned from the more classical force field methods. The “rigid skeleton”, the pores of the POCs, and host–guest interactions are of considerable interest and continue to be investigated.^70^ The total number of atoms in a given POC is often greater than 100, for which the high-level wave function methods are too expensive. Therefore, electronic structure calculations for POCs commonly rely on density functional theory (DFT), which gives more reliable results with acceptable simulation costs. Recently, computational simulations for extended porous frameworks have witnessed significant developments—in particular, when predicting their gas adsorption^71^ and diffusion properties.^72^ The wealth of new data, however, is not immediately useful when we address the applications of POCs. Consider the following: (a) How can we quantify the structural response of hosts toward guest molecules, since most organic molecular cages undergo swelling, structural rearrangement or phase changes upon the insertion of guests? (b) How about the bulk property response to the same structural impulse? (c) What about the effects of specific solvents on the interaction, since small changes in the angle or vertex of the organic linker precursors can affect cage geometry and porosity dramatically? (d) How about the weak interactions between the discrete molecular cages in their solid-state? (e) How about the position of the disordered solvent molecules located within the prefabricated pores? In response to these questions, computational methods have been extended to the field of POCs with a few recent examples^73−83^ that have been successful.

**Figure 4:**
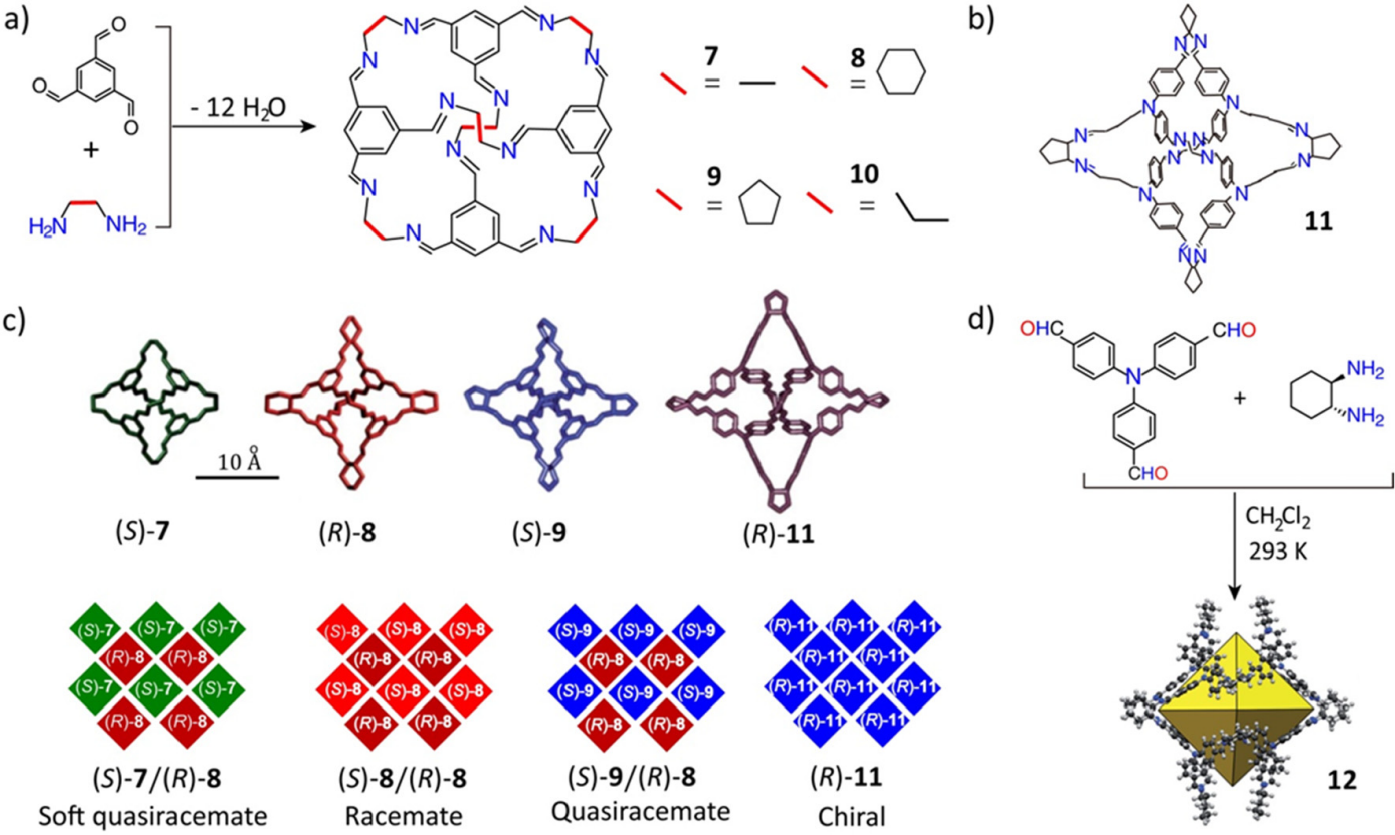
(a) Synthesis of imine-linked cages **7**, **8**, **9**, and **10** and their structural formulas. Reproduced with permission from ref (73). Copyright 2011 Springer-Nature. (b) Structural formula for the imine-linked cage **11**. Reproduced with permission from ref (74). Copyright 2014 The Royal Society of Chemistry. (c) Binary cocrystals of different chiral cages. Reproduced with permission from ref (73). Copyright 2011 Springer-Nature. (d) Synthesis of an imine-linked cage **12**. Reproduced with permission from ref (53). Copyright 2011 Wiley-VCH.

The crystallographic structure prediction of POCs can reveal^51^ their energetically favorable conformations, crystal packing arrangements, and pore sizes. Such predictions involve the optimization of a single molecular structure through electronic-structure calculations, while neglecting the influence of disordered solvent molecules. A prominent example^73,74^ is a series (Figure 4a,b) of [4 + 6] imine-linked cage crystals and cocrystals. Distinct chiral molecular cages have been self-assembled as a result of the global lattice energy minima of the conformations being incorporated into the calculated preferences, including heterochiral [(*S*)-**7**/(*R*)-**8** and (*S*)-**8**, (*R*)-**8**] and homochiral (*R*)-**11** packing arrangements. Crystal structure prediction has proved that the different forms of cage **7** have similar simulated lattice energies for their racemic and enantiomeric packing arrangements, an observation which could be used to rationalize the fact that cage **7** can be readily interconverted between different polymorphs in the solid state. As reflected in the large energy gaps between the lowest-energy predicted racemic and enantiomeric conformations, cages **8** and **9** prefer to form racemic crystals with heterochiral window-to-window packing, and cage **11** prefers to form homochiral crystals also with window-to-window packing (Figure 4c). In addition to conformation, the prediction of stoichiometry and pore size of cages is also possible on the basis of the structures of the organic linker precursors. One group have reported^75^ that the lowest energy calculations of the odd–even alternations of a series of possible [2 + 3] and [4 + 6] imine cages were well matched their experimental counterparts—namely, stoichiometry, size, and odd–even preferences while increasing the length of the alkane diamine chain. In another report,^76^ the crystal packing preferences of imine cages with additional methyl groups inside their windows were predicted: the introduction of methyl groups narrowed their pore sizes and affected their crystal packing arrangements. Consistent with the calculated CSP results, two types of experimental imine-linked cages prefer to form window-to-window and window-to-arene packing,^76^ respectively, depending on the position of methyl groups in the aldehyde linker molecules.

In reality the complexity of simulating POCs arises from the fact that host–guest interactions are dynamic processes. Considering the high flexibility of cage skeletons, host–guest interactions play a prominent role in the porosity of the POCs. In a recent study, Holden et al.^77^ classified the porosity in porous molecular solids into the following three types: (1) static porosity—the molecular skeletons are rigid, and there is no change in the structure when the guest molecules pass through the pores, (2) dynamic porosity—although the molecular skeletons are flexible, the porosity is intrinsic rather than rationalized by the movement of the guest molecules, and (3) cooperative porosity—the molecular skeletons are flexible, and the temporary porosity is rationalized predominantly by the host–guest interactions. The computational simulation approach was found to be the best way to distinguish between these three types of porosity and understand their underlying dynamic phenomena. Molecular dynamic simulations predict the movements between the cages and solvent molecules, the host–guest energies, and the impact of the guest molecules on the cage structures. For instance, the molecular dynamic simulations rationalized^78^ the “ON–OFF” porosity switching of cage **7** for H_2_ uptake, while describing a mechanism in which H_2_ molecules have a relatively short residence time when passing through the channels of organic molecular cages. In order to investigate this unusual behavior, a combination of *in situ* powder X-ray diffraction, gas sorption isotherms, and molecular dynamic simulations was used to study^77^ the porosity of another imine-linked cage, **10**. The cavities of cage **10** exhibited dynamic porosity to H_2_ molecules and cooperative porosity to Xe and CO_2_ molecules, while its one-dimensional (1D) pore channels exhibited static porosity to all three gases.

Since topology may influence physicochemical properties, molecular simulations have also proved useful in predicting the topological possibilities of POCs. In an example^79^ for calculating the most probable topology, the molecular dynamics simulations predicted numerous lowest-energy topologies for POCs, which were all then confirmed with wet laboratory experiments. In a recent study,^53^ molecular dynamics simulations indicated that the imine cage **12** undergoes a structural collapse to a nonporous form, a result which matches (Figure 4d) well with experimental observations. In addition, by calculating the host–guest interaction energies,^80^ molecular dynamics simulations also predict the diffusion mechanisms and selectivity for C8 aromatics in POCs.

Since wet synthetic approaches cost a lot of time and money, computational approaches are vital in any high-throughput screening of target molecular cages. What usually happens is that a great many products are formed by multiple organic linkers in one-pot reactions. Recently, a high-throughput discovery for new POCs was accomplished^81^ by theoretical simulations—based on geometry optimization and high-temperature molecular dynamics simulations—and consolidated with robotic synthesis and real-time characterization techniques. The authors found that reduction of symmetry plays a critical function when it comes to improving the porosity and solubility of the resultant POCs. The computational screening of 10,000 combinations of multidentate aldehydes and amines^82^ directed the successful synthesis of microporous, highly soluble, and unsymmetrical cages. In a more recent investigation,^83^ inspired by the concept of cage discovery from robotic automation of reactions, an optimized computational screening algorithm was developed to predict whether a molecular cage can encapsulate small molecules while preventing them from escaping. Although force field computational simulations and the electronic structure calculations can save several months of laboratory synthesis, they are still time-consuming and challenging, especially for molecules with over one hundred atoms. Machine-learning techniques could solve this problem and provide a fast-screening method for large molecules, along with accurate predictions for shape-persistence, pore size and window symmetry. In a recent report,^84^ machine learning showed progress by taking only a few milliseconds to predict these properties, based on a large database with 60,000 cage molecules. We must note, however, that the most-reported simulated prediction methodologies have focused on the fabrication of POCs using dynamic covalent chemistry, for the simple reason that it is more tractable for algorithms to predict the structures of thermodynamic products compared to kinetic ones. Consequently, new computational prediction theories and methods are highly anticipated for POCs that form as kinetic products.

## 3 Synthesis of Porous Organic Cages

The fabrication of POCs can occur through the following two distinct bond-forming routes: (i) irreversible linking chemistry and (ii) dynamic covalent chemistry (DCC). In this Section, we describe numerous representative attempts to assess the pros and cons of these two synthetic pathways.

### 3.1 Irreversible Bond-Forming Chemistry

Nonequilibrium, irreversible chemical transformations can furnish excellent chemical and thermal stabilities to the structures of POCs, while featuring limited functionalities. Since the formation of mechanically interlocked structures through strong chemical coupling reactions are rather uncommon,^22^ the discovery of novel organic molecular cages by irreversible linking chemistry is quite valuable and continues to be practiced for fundamental reasons. There is still a significant gap, however, between the inevitably low yields and bulk-scale industrial applications. We anticipate that this problem will be resolved in the near future by advances in synthetic chemistry, laboratory equipment, and molecular simulations.

#### 3.1.1 Amide Bond Formation

Amide bond formation is undoubtedly one of the most promising synthetic routes for making POCs as a consequence of its established synthetic toolbox, high rigidity of the adducts, and resonance-locked, limited bond rotation. Typically, the synthesis of an amide-linked cage involves cyclization of the prefabricated, hemispheric organic linker precursors under high dilution. A hexalactam cage **1a** with excellent solubility in chloroform was reported^47^ (Figure 1) in early 1984, using an amidation route in which 1,3,5-benzenetriyltris(methaneamine) and a tris(acid chloride) were reacted under high dilution—the yield (13%), however, was poor. The methoxy groups present in this cage were modified by using boron tribromide in dichloromethane to give the hexahydroxy cage **1b**.

**Figure 5:**
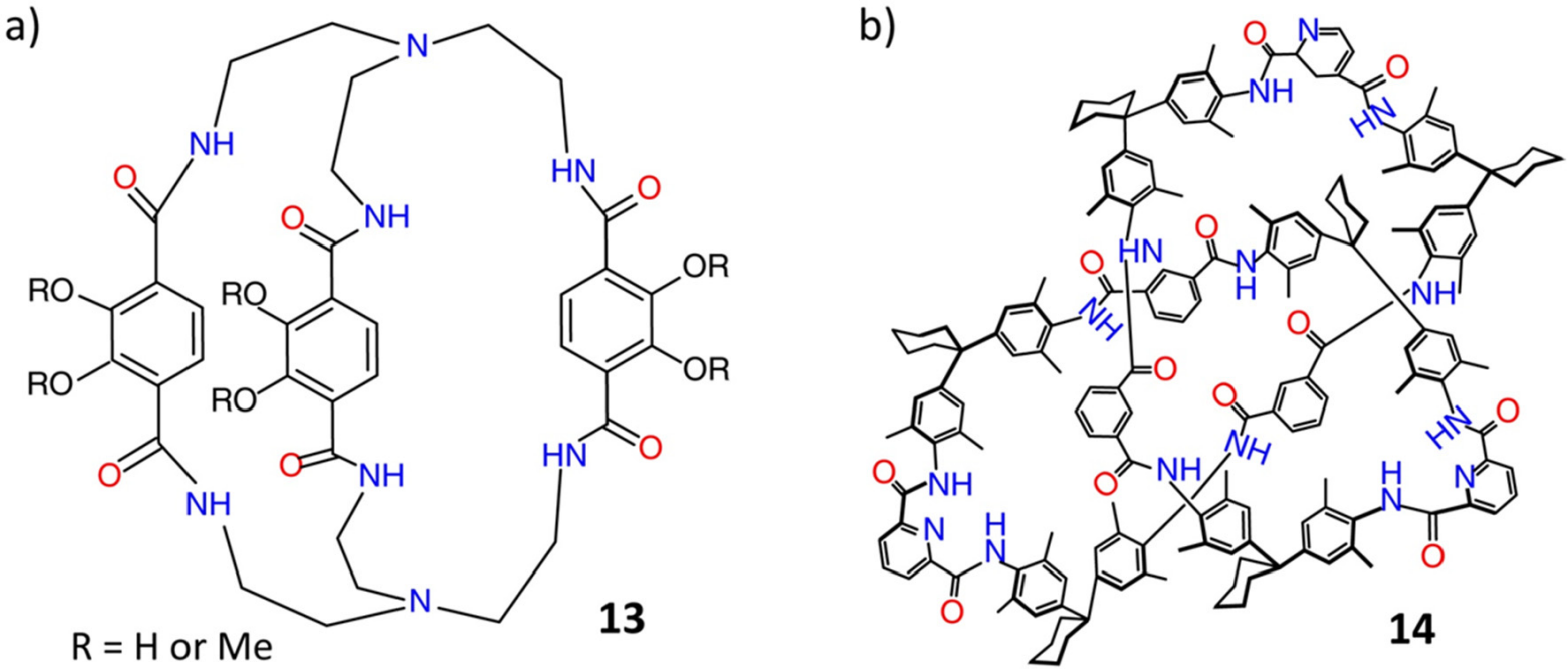
(a) Structural formula of bicapped cage **13**. Reproduced with permission from ref (85). Copyright 1987 American Chemical Society. (b) Structural formula of trefoil knot **14**. Reproduced with permission from ref (86). Copyright 2000 Wiley-VCH.

Later on, using the same approach, the bicapped cage compound **13** was synthesized^85^ (Figure 5), and its size, shape, and yield were found to be akin to the hexalactam cage **1a**. Furthermore, to overcome the flexibility of noncyclic organic linkers, a molecular trefoil knot **14** was obtained^86^ following the reaction (Figure 5) of 2,6-pyridinedicarboxylic acid dichloride and 1,1-bis(4-amino-3,5-dimethylphenyl)cyclohexane. Here, intramolecular hydrogen bonding between the amide groups is believed to assist product formation.

**Figure 6:**
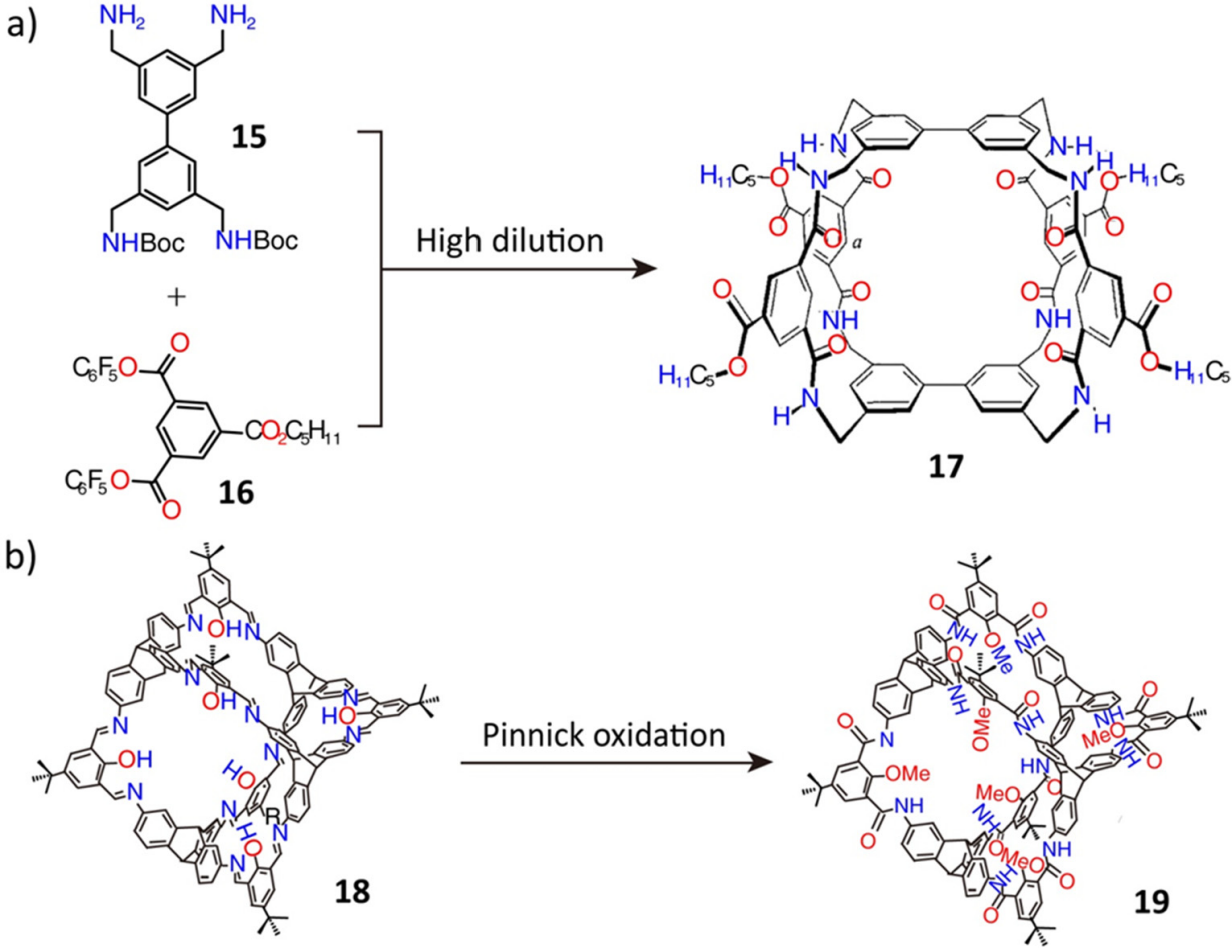
(a) The synthesis of the tricyclic polyamide cage **17**. Reproduced with permission from ref (88). Copyright 1998 Wiley-VCH. (b) The final step in the synthesis of amide cage **19**. Reproduced with permission from ref (59). Copyright 2019 Wiley-VCH.

It has been shown^85−94^ that the introduction of transition metal cations as templates and chemical tuning of the precursors can overcome the low-yields of the amide-linked cages. For example, the yields of bicapped cage^85^**13** and the amino acid bridged cage^87^ were elevated to 70% by metal-template-driven multicomponent cyclizations at high dilution. Likewise, inspired by the carbohydrate-bonding proteins, tricyclic polyamide cage **17** was prepared^88^ in a low yield that was attributed (Figure 6a) to the poor final cyclization of the asymmetrically protected biphenyl intermediate **15** and bispentafluorophenyl ester **16**. The yield was improved^89^ (62%) by simply modifying the −OC_5_H_11_ functional groups in the corresponding biphenyl and isophthaloyl precursors. In another example,^90^ the yield of a helical amide-based cage was enhanced significantly by using *m*-diaminobenzene rather than the *p*-substituted isomer. It is worth mentioning that amide-linked cages with small molecular structures can be synthesized efficiently by one-step cyclizations of their prefabricated precursor oligomers. For instance, small chiral spherical molecular cages have been cyclized^91^ by employing preorganized aromatic amide components in a yield of 56%. Similarly, one-step reactions of some rigidified and suitably sized oligomers have led to the formation of shape-persistent aromatic oligoamide macrocycles^92,93^ and circular aromatic pentamers.^94^ The reactions of the amide bonds are predominantly kinetically controlled, a feature that makes it difficult to construct in high yield amide-linked cages with extensive molecular frameworks. As an alternative, a Pinnick oxidation approach^59^ was developed recently. A high-yield salicylbisimine cage **18**, which was prefabricated by reversible DCC and subsequently oxidized to afford the robust amide cage **19**, is a good example (Figure 6b) of locking-in a large molecular geometry.

#### 3.1.2 Carbon–Carbon Bond Formation

When it comes to irreversible strong bonding, C–C bonds take the lead because of their inherent covalent nature and substituent versatility, while maintaining π-electron conjugation. In addition, a complete C–C bonded framework with unaltered conjugation can endow porous organic molecular cages with photochemically active functional groups.^95^ The challenge, however, lies in the fact that the C–C linkages favor the formation of linear polymers rather than cages, if the kinetic parameters are not controlled. Consequently, C–C bond formation routes often afford cages in low yields.

**Figure 7:**
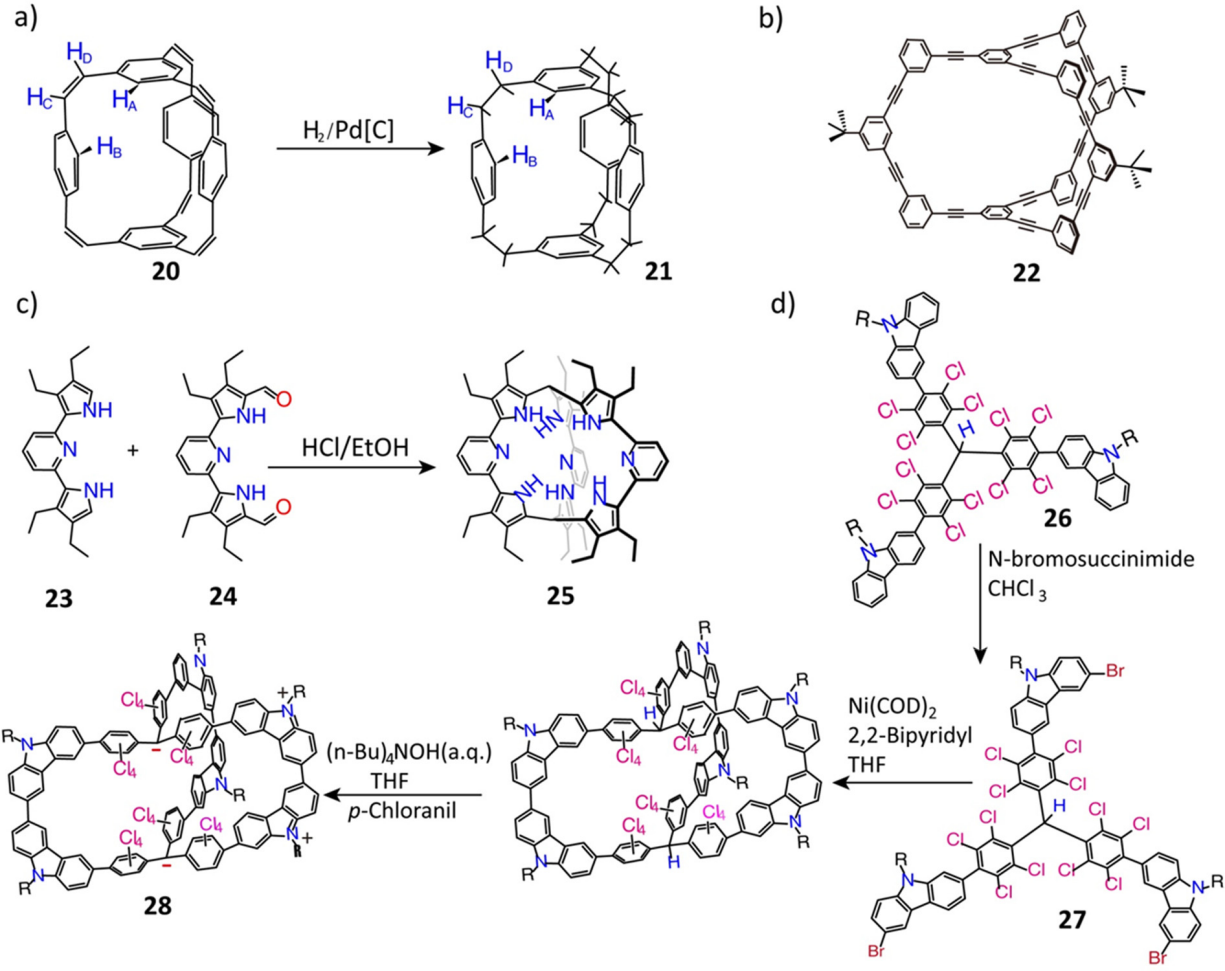
(a) Synthesis of the bicyclophane cages **20** and **21**. Reproduced with permission from ref (46). Copyright 1977 Elsevier. (b) Synthesis of *D*_*3h*_ symmetric triangular prism cage **22**. Reproduced with permission from ref (99). Copyright 1992 American Chemical Society. (c) Synthesis of calixpyrrole-like cryptand **25**. Reproduced with permission from ref (100). Copyright 2001 American Chemical Society. (d) Synthesis of 3D conjugated cage **28**. Reproduced with permission from ref (101). Copyright 2017 Wiley-VCH.

For example, the bicyclophane cage **20** with an all-carbon skeleton^46^ (Figure 7a) was synthesized by a one-step Wittig reaction between 1,3,5-benzenetricarbaldehyde and 1,4-bis(bromomethyl)benzene in a dry dimethylformamide (DMF) solution. The cage was isolated in the low yield of 1.7%. This bicyclophane cage **20** was reduced to the saturated bicyclophane cage **21** by hydrogenation using palladium as the catalyst. In subsequent work, multistep condensation techniques were introduced to fabricate molecular cages composed of trinacrene,^96^ heptacyclics,^97^ and concave hydrocarbons,^98^ with only slight improvements (2%) in the yields.

In subsequent years, optimized catalysts were shown^99−101^ to promote the formation of C–C linked cages. A real breakthrough came with (Figure 7b) the synthesis^99^ (achieved by a double-cyclization method using KOH and [Pd/(dba)_2_]/Ph_3_P/CuI) of the *D*_*3h*_ symmetric triangular prism cage **22** in an overall yield of 32%. A Brønsted acid-catalyzed calixpyrrole-like cryptand **25** was obtained^100^ (Figure 7c) in 48% yield from the corresponding bipyrrole **23** and dialdehyde **24**, akin to the syntheses of calixarenes or pillarenes. The pyrrolic NH group facing the cage interior renders **25** promising for binding small molecules. In addition, in the presence of Ni(COD)_2_/2,2′-bipyridyl, a 3D π-conjugated molecular cage **28** (Figure 7d) was prepared^101^ in 10% overall yield by intermolecular Yamamoto homocoupling of the tribromide **27** followed by deprotonation and oxidation. Tribromide **27** was prepared in 77% yield by a regioselective bromination of the key intermediate, **26**, which was obtained in 90% yield by Suzuki coupling of tri(4-iodo-2,3,5,6-tetrachlorophenyl)methane and 9-*n*-butyl-*9H*-carbazol-3-yiboronic acid using Pd(PPh_3_)_4_ as a catalyst.

**Figure 8:**
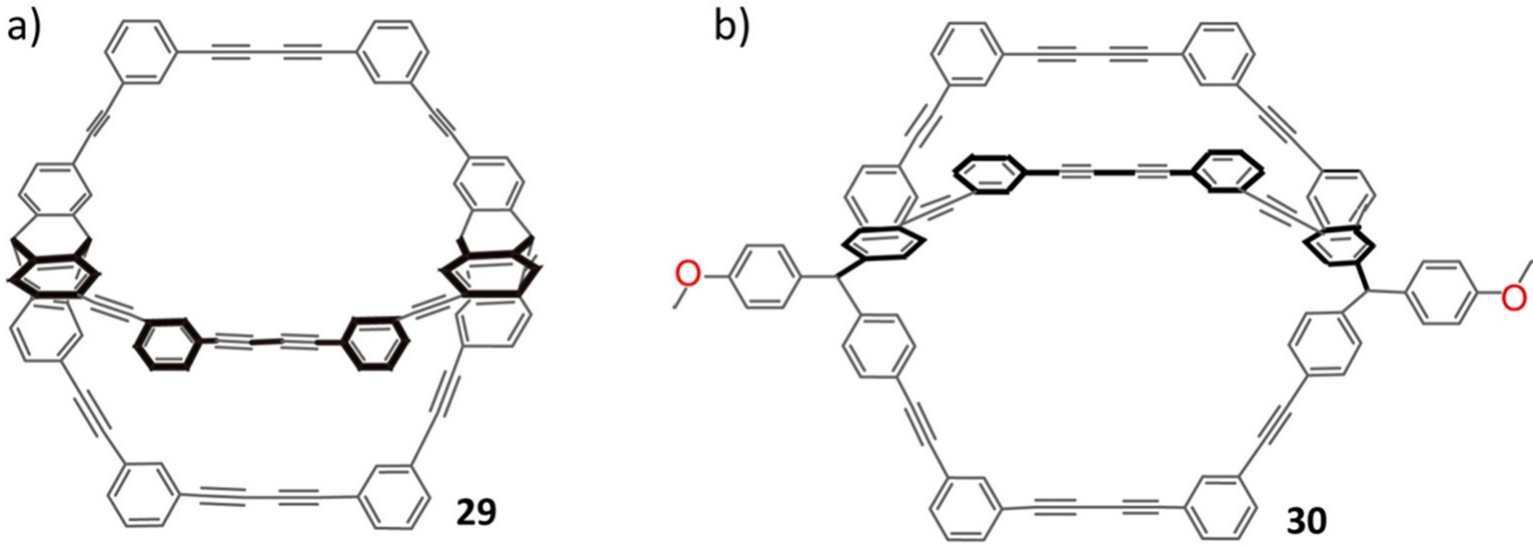
Structural formulas of the triptycene-based cages **29** and **30**. Reproduced with permission from ref (102). Copyright 2007 American Chemical Society.

The rigidity and angular positioning of the building blocks have a considerable influence^102,103^ on the yield of the C–C linked cages. A good example^102^ of these criteria at work is the construction of triptycene-based molecular cages through a copper-mediated Eglinton–Glaser coupling. The terminal acetylene linkers with two methine units are more rigid than the linker without the methines. The triptycene-based molecular cage **29**, however, synthesized (Figure 8) from the terminal acetylene linkers without the two methine units, gave a much higher yield (58%) than that (20%) for the molecular cage **30** synthesized from the terminal acetylene linkers bearing two methine units. The triptycene-based molecular cage **29**, prepared from the terminal acetylene organic linkers without methine units, has two kinetically controlled polymorphs. Despite considerable effort, the one-step synthesis of C–C bond-linked cages in high yield has yet to be realized. Cyanostar,^103^ a cyanostilbene-based macrocycle with *C*_5_-symmetry, is a case in point: it was obtained by the Knoevenagel condensation in 81% yield. In the mixed apolar-protic solvents, the enantiomers of cyanostars form sandwich complexes because of their shallow bowl shape and the electron-deficient cyanostilbene units. The 3D cage-forming versions of this kind of traditional chemistry might enable high yields of C–C bonded POCs.

#### 3.1.3 Nucleophilic Substitutions

**Figure 9:**
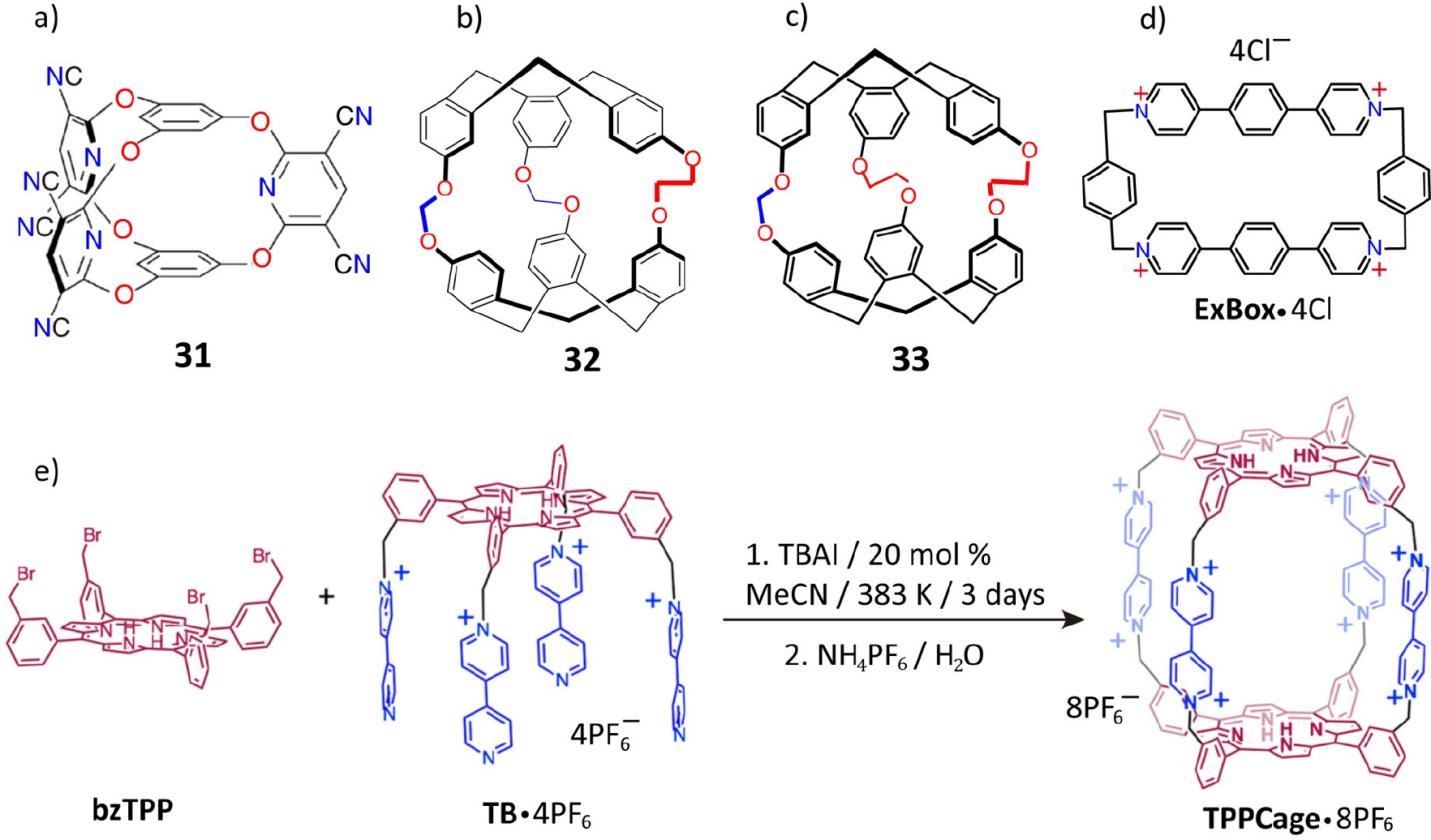
(a) Structural formula of the bicyclooxacalixarene **31**. Reproduced with permission from ref (104). Copyright 2005 American Chemical Society. (b) Structural formula of the cryptophane cage **32**. Reproduced with permission from ref (105). Copyright 2010 American Chemical Society. (c) Structural formula of the cryptophane cage **33**. Reproduced with permission from ref (106). Copyright 2011 American Chemical Society. (d) Structural formula of the semirigid cyclophane **ExBox**^**4+**^. Reproduced with permission from ref (112). Copyright 2013 American Chemical Society. (e) Synthesis of the tetragonal prismatic porphyrin cage **TPPCage**^**8+**^. Reproduced with permission from ref (114). Copyright 2018 American Chemical Society.

Stable, irreversible covalent bonds can also be formed through the nucleophilic substitutions^500^ and, therefore, used to construct novel POCs. An example is small organic molecular cages featuring C–O bonds. By applying the cesium cation as a template,^104^ the condensation of phloroglucinol with an electron-poor pyridine led (Figure 9a) to the formation of the cage-like bicyclooxacalixarene **31** in 95% yield. In another instance,^105,106^ coupling of ethylene glycol bis-tosylate and cyclotriphenolene linkers led to stable intermediates, which were cyclized with bromochloromethane to form two cryptophane cages, **32** and **33**, with a mixture of methylene (Figure 9b) and bismethylene (Figure 9c) links.

The introduction of C–N^+^ bonds as routine building-block linkages^107,108^ by one of us has led to the design and development of robust organic molecular cages. A series of molecular cages with varying geometries, such as the Blue Box,^109^ a tetracationic molecular receptor,^110^ an X-shaped octacationic cyclophane^111^ (XCage^8+^), a semirigid cyclophane^112^ (**ExBox**^**4+**^), a hexacationic triangular covalent organic cage^113^ (AzaEx^2^Cage^6+^), and a tetragonal prismatic porphyrin cage^114^ (TPPCage^8+^), have all been made and characterized. For the sake of brevity, we will feature only the two classical examples (Figure 9d), **ExBox**^4+^ and **TPPCage**^8+^. **ExBox**^4+^, a robust tetracationic organic cyclophane,^112^ was synthesized from 1,4-phenylene-bridged bipyridine and bisbromomethylbenzene. Using pyrene as a template, the cyclization afforded a yield of 42%. **TPPCage**^8+^ was obtained^114^ in 19% yield from **bzTPP** and **TB·4PF**_**6**_ (Figure 9e) in the presence of tetra-*n*-butylammonium iodide (TBAI). The entire synthetic approach involved rapid S_N_2 nucleophilic substitutions in three steps from 3-(bromomethyl)benzaldehyde, pyrrole, and **TB·4PF**_**6**_ formed from **bzTPP**.

### 3.2 Dynamic Covalent Chemistry

Dynamic covalent chemistry (DCC) entails the inherent error-correction mechanism of reversible chemical bonds to form thermodynamically stable POCs. In contrast with the irreversible-linking chemistry, DCC can lead to construction of molecular cages in higher yields from relatively simple organic linker precursors during one-pot reactions, while avoiding complicated purification procedures. Herein, we have described the most effective current approaches^115^ employing DCC, such as imine condensation (Schiff base chemistry), boronic ester condensation, and olefin/alkyne metathesis.

#### 3.2.1 Imine Condensation

Schiff base chemistry, the formation of imine bonds through the reversible condensation of amines and aldehydes, is one of the most commonly used routes to investigate the modular construction of POCs of different sizes, geometries, and functions. The construction of organic molecular cages by imine condensations dates back to 1991, when Donald Cram reported^116^ a shape-persistent molecular container in 45% yield by a [2 + 4] reaction of a tetraformylcavitand and 1,3-diaminobenzene. Three pioneering pieces of research reported^48−50^ in 2008 and 2009 led to the rapid development of Schiff base-linked POCs. These investigations involved the synthesis of (i) an imine cage by a [4 + 6] condensation of triptycene triamine and salicyldialdehyde,^49^ (ii) an imine tetrahedral cage **8** with permanent porosity by a [4 + 6] condensation of (*R*,*R*)-1,2-diamino-cyclohexane and 1,3,5-triformylbenzene for the purpose of gas adsorption,^50^ and (iii) a nanoscale chiral tube by a [8 + 12] condensation of a bowl-shaped aldehyde and *p-*phenylenediamine in 90% yield.^48^ Since these early investigations—mainly driven by the emergence of advanced characterization techniques and the need for porous materials to address current technological challenges—a large number of novel POCs have been obtained by imine condensation.^21,22^ At present, most investigations focus on optimizing precision syntheses toward large, highly porous, and stable molecular cages.^23,65^

**Figure 10:**
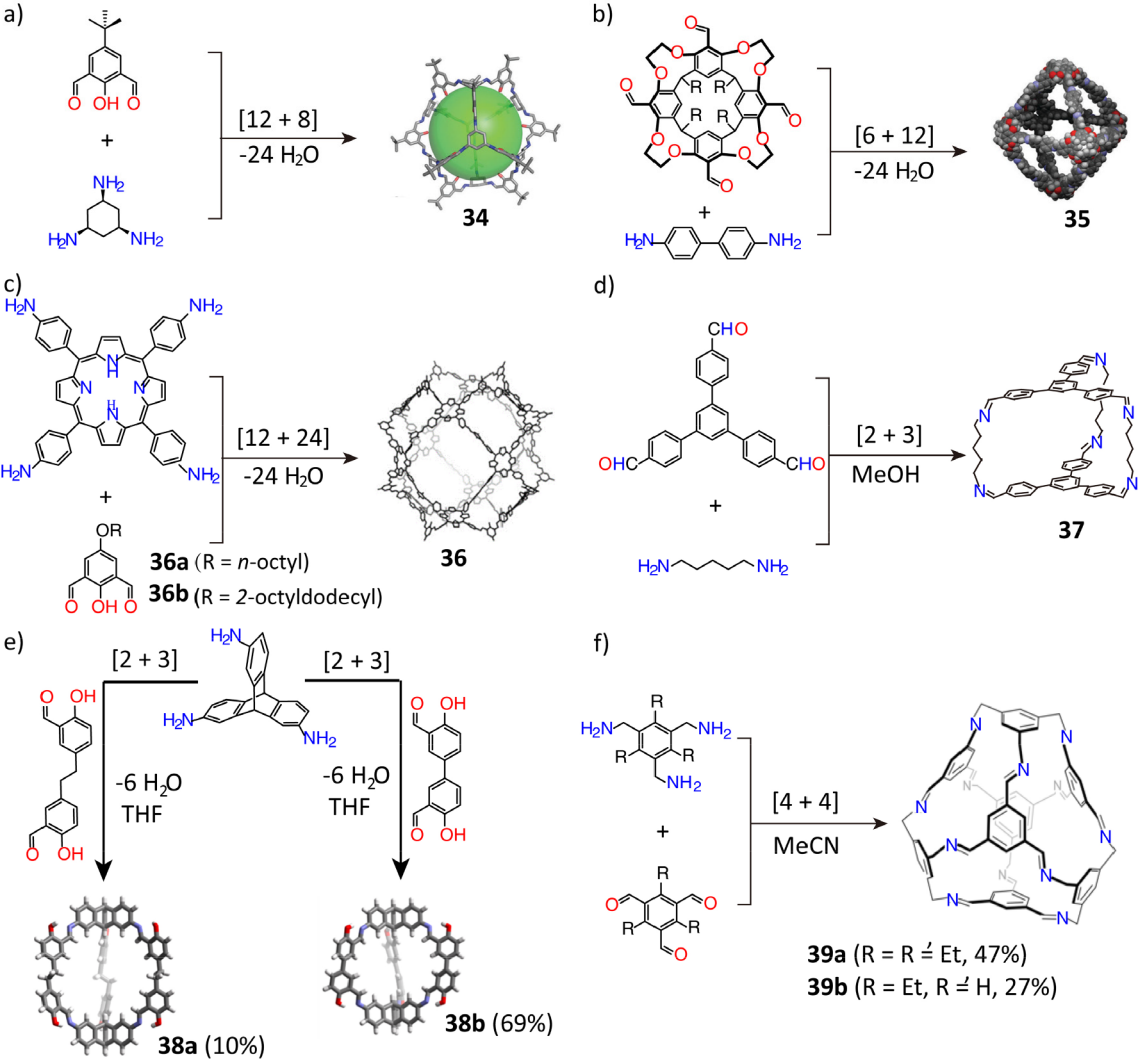
(a) Synthesis of a molecular cage **34**. Reproduced with permission from ref (117). Copyright 2013 The Royal Society of Chemistry. (b) Synthesis of molecular cage **35**. Reproduced with permission from ref (118). Copyright 2011 The Royal Society of Chemistry. (c) Synthesis of molecular cage **36**. Reproduced with permission from ref (119). Copyright 2020 Elsevier. (d) Synthesis of molecular cage **37**. Reproduced with permission from ref (120). Copyright 2011 The Royal Society of Chemistry. (e) Synthesis of molecular cage **38**. Reproduced with permission from ref (121). Copyright 2012 Wiley-VCH. (f) Synthesis of molecular cage **39**. Reproduced with permission from ref (122). Copyright 2018 Wiley-VCH.

In general, the use of extended organic linkers leads to the construction of large shape-persistent organic molecular cages. An elegant example^117^ of this reaction is the condensation of 4-*tert*-butyl-2,6-diformylphenol and 1,3,5-triaminocyclohexane, leading to the formation of a large [12 + 8] cage (**34**) with an outer diameter of 3.0 nm, as confirmed (Figure 10a) by single-crystal X-ray diffraction analysis. In addition, geometry-induced entropy can facilitate the formation of large symmetry cages. The [6 + 12] condensation of tetraformylcavitand with rigid, linear benzidine leads^118^ to the formation of a polyimine octahedral cage **35**, which has a large solvodynamic diameter of 4.68 nm, as confirmed (Figure 10b) by diffusion ordered spectroscopy (DOSY) and nuclear magnetic resonance (NMR) spectroscopy. Recently, it was reported^119^ that a gigantic, record-breaking organic cage (**36**) with a cuboctahedral geometry can be constructed in 17% yield by a [12 + 24] condensation of the square-shaped tetra(4-aminophenyl)porphyrin and bent-shaped 2-hydroxy-5-octyloxy-1,3-benzenedicarboxaldehyde in dry 1,2-dichlorobenzene. Powder X-ray diffraction analysis (Figure 10c) confirmed that the cage has an outer diameter of 5.3 nm.

High flexibility and rotational freedom in the extended chains of organic linkers can disrupt the formation of large cages. In one report,^120^ the reaction of an extended 1,3,5-tri(4-formylphenyl)benzene with 1,5-pentanediamine afforded the small [2 + 3] propeller-shaped cage **37** with 1D channels instead of the larger [4 + 6] imine cage, a result (Figure 10d) that can be attributed to high flexibility in the 1,5-pentanediamine linkers. In another report, a large chiral cage, **12**, with an outer diameter of 2.9 nm was constructed^53^ by an [8 + 12] condensation of tris(4-formylphenyl)amine and the chiral (*R*,*R*)-1,2-cyclohexanediamine. This cage, however, collapsed after the removal of the guest solvents, as a consequence of unrestricted rotations about the C_arene_–C_arene_–C_imine_–N_imine_ torsion angles at the cage vertices, and the C_arene_–C_arene_–N_amine_–C_arene_ torsional angles located on the trialdehyde surfaces of the cage. Consequently, a critical question is how much the rigidity of organic linkers influences the formation of shape-persistent imine-linked cages. In this context, the condensation of triptycene triamine with two different bis(salicylaldehyde) linkers was investigated^121^ (Figure 10e), where one of the bis(salicylaldehyde) linkers, possessing an ethylene bridge between the two salicylaldehyde units, has a higher flexibility than the other one. The cage product **38a** formed with the flexible bis(salicylaldehyde) linker was obtained with a lower yield of 10%, while the cage product **38b**, based on the rigid bis(salicylaldehyde) linker, was isolated in a much higher yield of 69%. In other work, the influence of the rigidity of organic linkers on the formation of a truncated tetrahedral cage was investigated.^122^ The [4 + 4] condensation of a 1,3,5-triethyl-substituted triamine with two different aldehydes, namely 1,3,5-triformylbenzene and triethyl-substituted 1,3,5-triformylbenzene, led (Figure 10f) to the formation of the tetrahedral cages **39a** and **39b** in moderate yields of 47 and 27%, respectively. If, however, the triamine lacked ethyl substituents, the tetrahedral cages did not form because of the free rotation of benzylamine units around C–C bonds. It follows that the flexibility of the organic linkers must be taken into account prior to the construction of shape-persistent imine-linked cages.

**Figure 11:**
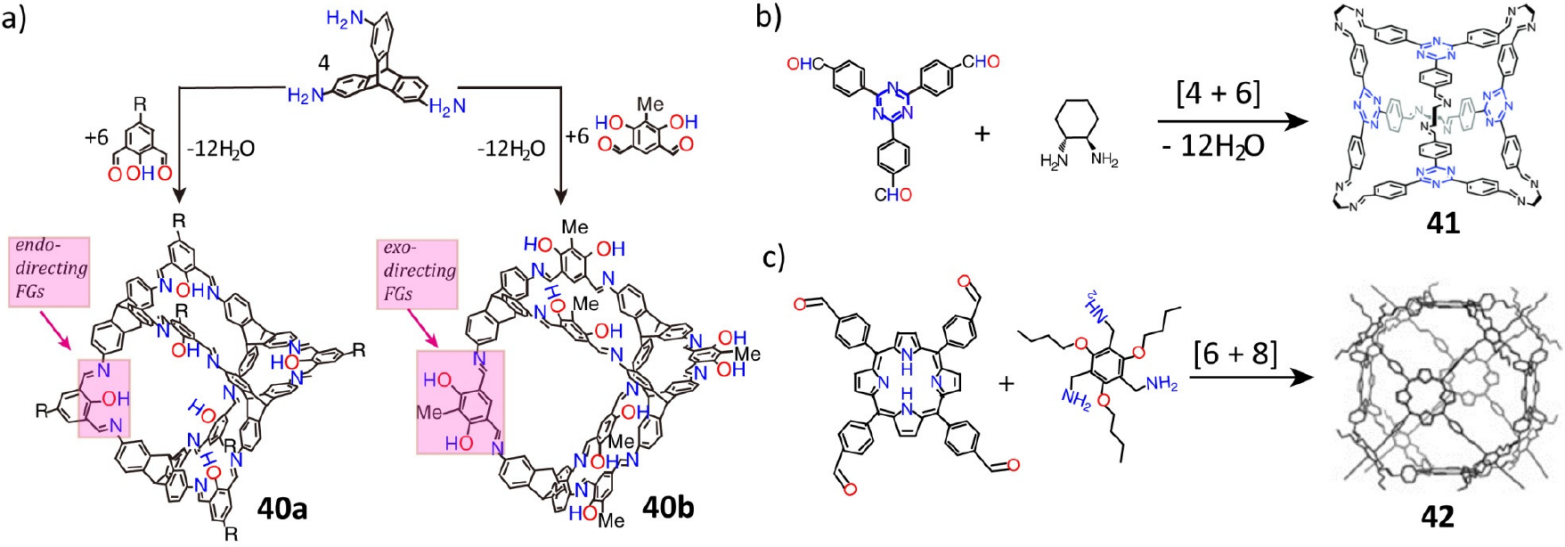
(a) Construction of molecular cages **40**. Reproduced with permission from ref (123). Copyright 2012 The Royal Society of Chemistry. (b) Construction of molecular cage **41**. Reproduced with permission from ref (125). Copyright 2015 The Royal Society of Chemistry. (c) Construction of molecular cage **42**. Reproduced with permission from ref (126). Copyright 2015 Wiley-VCH.

Substituents on the organic linkers play a key role in the formation of organic molecular cages. For instance, the [4 + 6] condensation of triptycene triamine with salicyldialdehyde and resorcinol dialdehyde has led^123^ (Figure 11a) to the synthesis of the endofunctionalized shape-persistent cage **40a** and the exofunctionalized cage **40b**, respectively. Moreover, if the *tert*-butyl group—at the para-position to the phenolic hydroxyl group—in the salicyldialdehyde linker is replaced by methyl group or hydrogen atom, the cage formation time is extended from 7 to 11 or 22 days. When the phenolic hydroxyl group is substituted by methyl, methoxy, or hydrogen,^124^ undesired side products are observed. These observations can be attributed to the stereoelectronic effects of the substituents, which can alter the electronic demands of the formyl groups.

A slight change in the geometry of the organic linkers also affects the formation of organic molecular cages. For example, depending on the chain length and number of carbon atoms, an odd–even effect is observed^75^ for the formation of [2 + 3] and [4 + 6] imine-linked cages. A [4 + 6] condensation of the electron-deficient triazine precursor^125^ with chiral amines has led (Figure 11b) to the construction of tetrahedral cage **41**. In another example, a [6 + 8] condensation of quadrilateral porphyrin derivatives and a triamine formed^126^ (Figure 11c) an Archimedes cubic-like cage (**42**) with a pore size of 1.93 nm in 99% yield. In addition, three types of cages, namely, [2 + 4] dimeric cages with odd–even behavior, [3 + 6] trimeric triangular prisms, and [6 + 12] hexameric octahedra, were formed^127^ by the condensation of the tetraformylresorcin[4]arene cavitand with different diamine linkers. Other factors influencing cage formation include the reaction solvents, temperature range, concentrations, and catalysts. For example, in the condensation of the same tetraformylcavitand and ethylene-1,2-diamine, the octahedral, tetrahedral and square-anti prismatic cages were obtained^128^ by simply changing the reaction solvents. In another example, the triply interlocked cages were afforded^67^ by the one-step condensation of a trialdehyde and different diamines catalyzed by trifluoroacetic acid (TFA) in acetonitrile without the use of an additional template, even though the noninterlocked, monomeric cages were formed^50^ in the same solvent in the absence of TFA.

**Figure 12:**
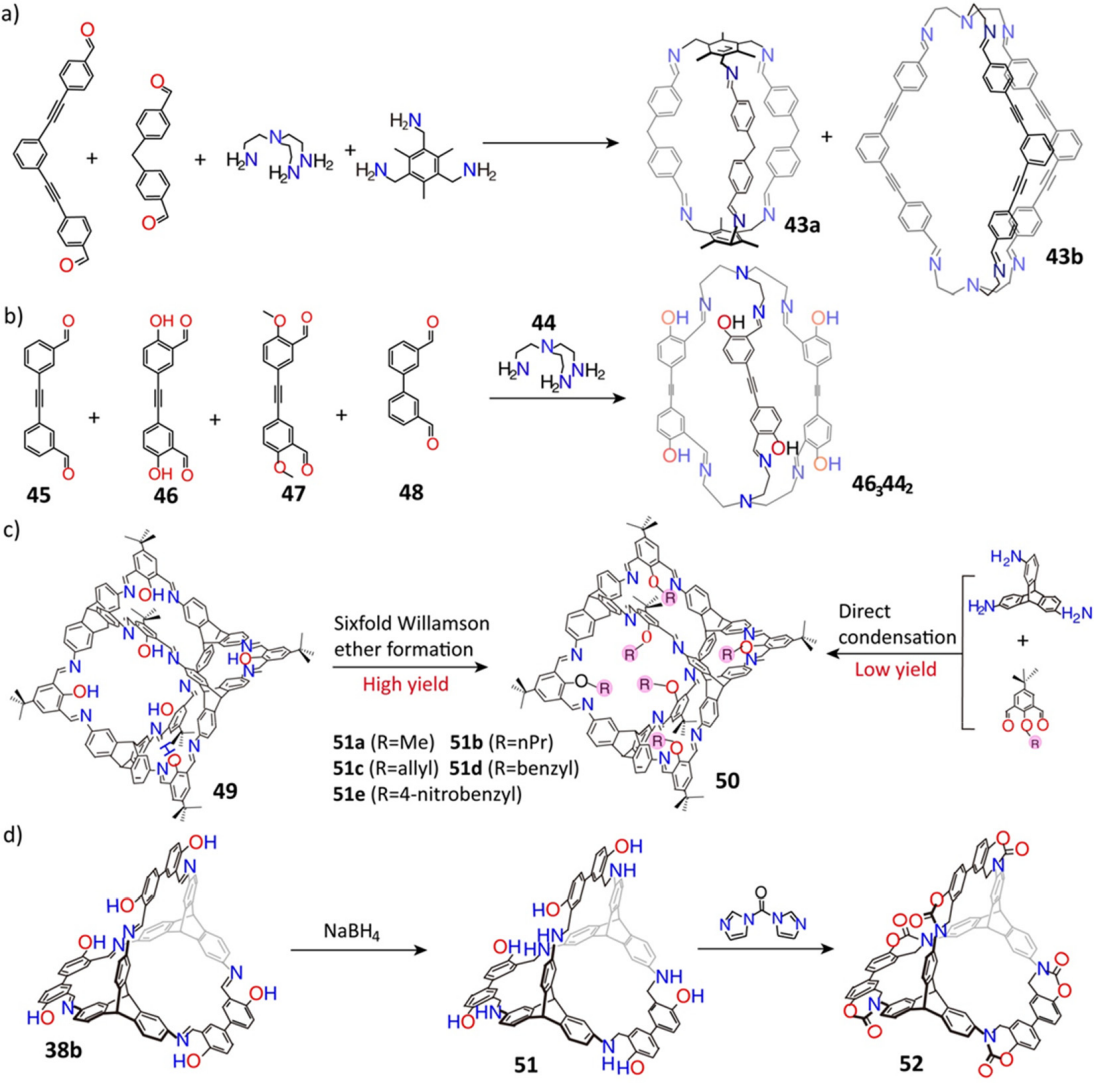
(a) Synthesis of molecular cages **43**. Reproduced with permission from ref (129). Copyright 2013 American Chemical Society. (b) Synthesis of molecular cage **46**_3_**44**_2_. Reproduced with permission from ref (130). Copyright 2014 Wiley-VCH. (c) Synthesis of molecular cage **50**. Reproduced with permission from ref (132). Copyright 2013 Wiley-VCH. (d) Synthesis of a molecular cage **52**. Reproduced with permission from ref (134). Copyright 2017 The Royal Society of Chemistry.

The dynamic nature of imine bonds also enables self-sorting among organic molecular cages. For example, the condensation of a solution of two iso-structural amines and a solution of two structurally similar aldehydes led (Figure 12a) to the exclusive synthesis of two types of cages, namely **43a** and **43b**,^129^ each comprised of one flexible and one rigid unit. In another, self-sorting (Figure 12b) was observed^130^ in the reaction of an amine (**44**) with four different aldehydes (**45**, **46**, **47**, and **48**), where only one type of cage **46**_3_**44**_2_ was obtained. If, however, a solution of amine **44** was reacted with a solution containing aldehydes **45** and **48**, two types of cages, **45**_3_**44**_2_ and **48**_3_**44**_2_, were generated. It was assumed that the hydroxyl groups on the aldehyde monomer **46** affect the self-sorting between amine and aldehydes. Moreover, by making use of self-sorting behavior during DCC, eight different organic molecular cages were produced^131^ by the condensation of constitutionally different aromatic aldehydes with one flexible amine. The selective formation of such different molecular cages relies highly on molecular flexibility, electronic factors, and the presence of a heteroatom in the organic linkers. In addition, the cages undergo catalytic dynamic component exchange in dilute acids.

Postmodification is another effective tool that is used in order to improve the yield and solubility of organic molecular cages. For instance, the hydroxyl groups in the [4 + 6] cage **49** were modified^132^ by the introduction of methyl, propyl, allyl, benzyl, and 4-nitrobenzyl groups (Figure 12c) in a 6-fold Williamson ether procedure, while maintaining high yields (63–81%) of their corresponding cages **50**. Direct imine condensation from substituted phenols, however, resulted in low yields and undesired products, particularly because hydroxyl groups facilitate the formation of cages. In addition, catenated imine cages with slight structural variations were self-assembled^133^ into a controlled hierarchy, leading to the formation of a number of superstructures.

Unfortunately, most organic molecular cages containing imine bonds are sensitive to moisture as well as acidic and alkaline conditions. The postmodification approach, however, exhibits great promise for obtaining highly stable imine-linked cages. In one report, the “tied” cage **6**, which exhibits high stability^64^ over the pH range 1.0–12.0, was constructed (Figure 3) by reacting the amine cage **5** and paraformaldehyde. Cage **5** was also prepared by the reduction of the chiral imine cage **8** using sodium borohydride. In another report,^134^ sodium borohydride reduction of a salicylimine cage (**38b**) led to the formation of amine cage **51**. The reaction of **51** with *N*,*N*-carbonyldiimidazole led to the formation of a shape-persistent porous carbamate cage **52** (Figure 12d), which has a remarkable stability in (i) concentrated HCl (pH = −1) at room temperature, and (ii) in 1 M HCl (pH = 0) at 100 °C. Acid tolerance of an organic molecular cage was also observed to be enhanced^135^ by efficient packing in the solid state of racemic, as opposed to chiral, forms where intermolecular mesopores in the solids allow faster degradation.

#### 3.2.2 Boronic Ester Condensation

**Figure 13:**
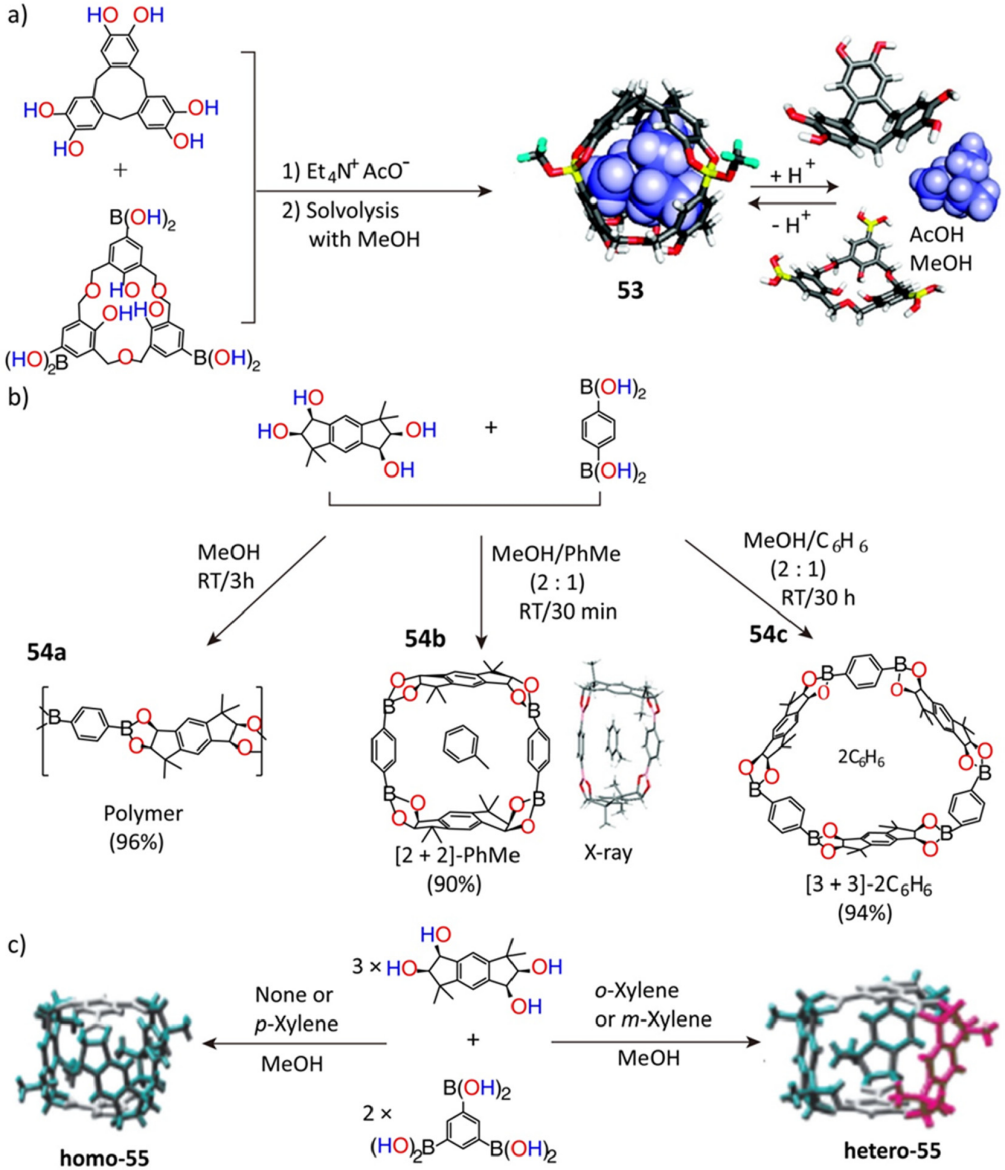
(a) Synthesis of the first boronic cage **53**. Reproduced with permission from ref (137). Copyright 2007 American Chemical Society. (b) Synthesis of the insoluble boronic polymer **54a**, macrocycle **54b**, macrocycle **54c**. Reproduced with permission from ref (138). Copyright 2007 American Chemical Society. (c) Formation of cages **homo-55** and **hetero-55**, through a change of solvents. Reproduced with permission from ref (139). Copyright 2009 Wiley-VCH.

The formation of boronic esters through a simple and reversible condensation of boric acids with catechols^136^ has generated considerable interest in their adaptation of the design and development of POCs. The first example, boronic cage **53**, was reported^137^ in 2007 as a result (Figure 13a) of a condensation of cyclotricatechylene and a boronic acid-appended hexahomotrioxacalix[3]arene with the addition of Et_4_NAcO. By simply changing the *p*Ka of the solvents, the decomposition and reconstruction of the isolated cages can be tuned thanks to the dynamic nature of the boronic ester linkages.

As in the case of the imine-based POCs, the synthetic conditions, and in particular the choice of guest molecules, influence the conformations of the boronic ester cages strongly. The condensation of a racemic tetraol containing two fixed 1,2-diol units and 1,4-benzenedi(boronic acid) with a planar structure affords^138^ (Figure 13b) three different kinds of products–namely, an insoluble boronic polymer **54a**, a [2 + 2] macrocycle **54b**, and a [3 + 3] microcycle **54c**, in three different solvents, i.e., methanol, methanol/toluene, and methanol/benzene, respectively. It was also shown^139^ (Figure 13c) in another study that using the same starting reagents—the racemic tetraol and 1,3,5-benzenetri(boronic acid)— but changing only the guest solvents can direct the formation of two types of boronic ester cages, namely, the symmetrical chiral boronic ester *homo*-[3 + 2] cage **55** in methanol or a mixed solvent of methanol and *p*-xylene, and the asymmetrical *hetero*-[3 + 2] cage **55** as a major product in the mixed solvents, such as *o*-xylene/methanol or *m*-xylene/methanol.

**Figure 14:**
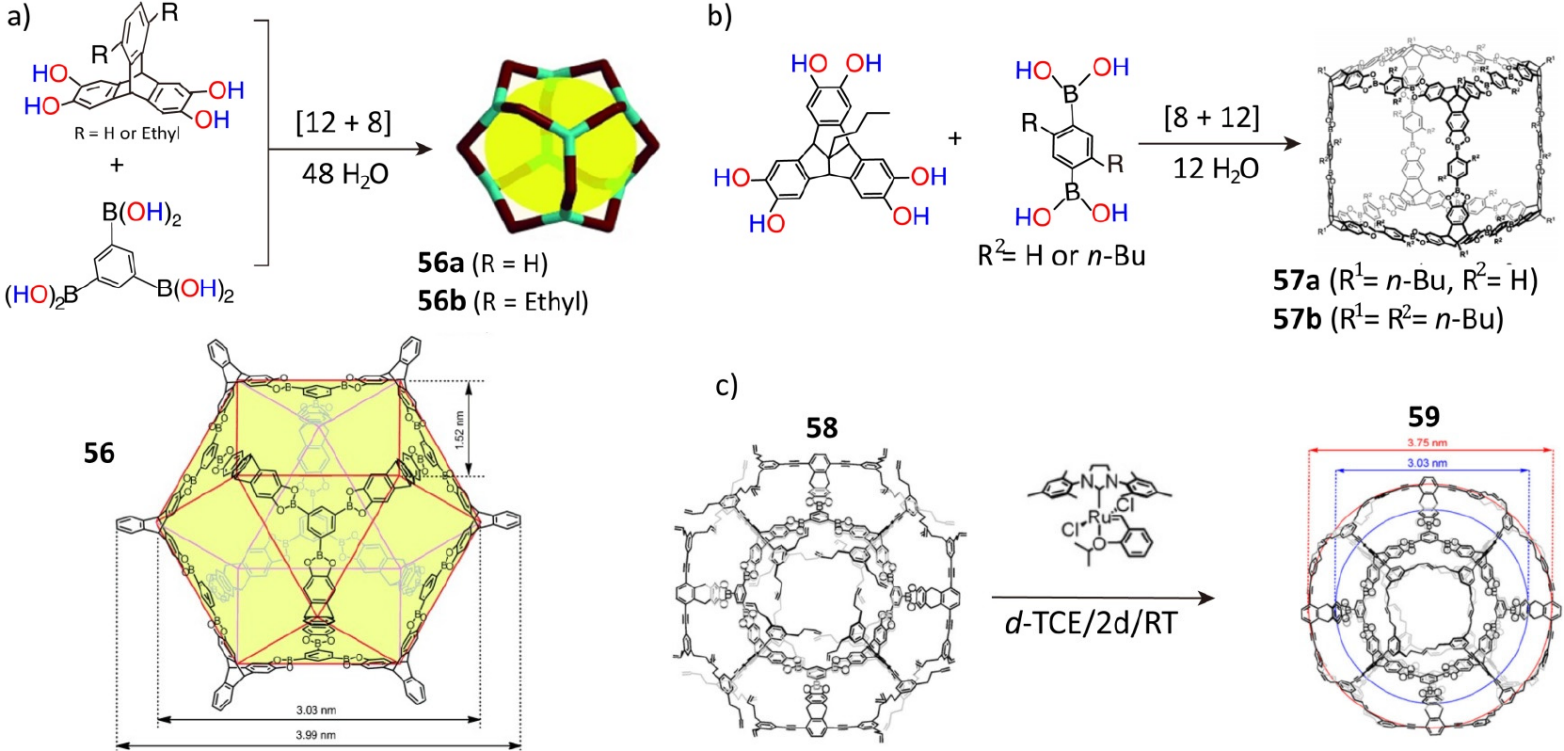
(a) Synthesis of cuboctahedral cage **56**. Reproduced with permission from ref (140). Copyright 2014 Wiley-VCH. (b) Synthesis of symmetrical cubic cage **57**. Reproduced with permission from ref (143). Copyright 2014 The Royal Society of Chemistry. (c) Formation of a giant cage **59**. *d*-TCE: deuteron-tetrachloroethane. Reproduced with permission from ref (146). Copyright 2021 Wiley-VCH.

The structures of boronic ester cages are also highly affected by the size or geometry of the organic linkers. The triptycene tetraol, with its 120° angle between the aromatic planes and the two ethyl substituents on the outer aromatic ring, is found (Figure 14a) to be an ideal linker, insofar as it reacts with 1,3,5-benzenetri(boronic acid) to form^140^ quantitatively a large cuboctahedral cage **56** with an outer diameter of 3.99 nm and an inner maximum diameter of 3.03 nm. Such cages are packed in a hexagonal network as a result of [π···π] stacking of the trisboronic ester units of adjacent cages in the solid state. If, however, a 9,10-dihexyltriptycene —consisting of long alkyl chains at its bridgehead positions— serves as a linker, then quadruply interlocked cage catenanes with ellipsoid shapes are formed^141^ during the crystallization. If the same alkyl chains are substituted by Br atoms on the triptycene linkers, condensation of the brominated hexaol triptycene with benzene 1,4-diboronic acids lead to the formation^142^ of tetrahedral [4 + 6] boronic ester cages. Perfluorination of diboronic acid accelerates the formation of boronic ester cages. The [8 + 12] condensation of the catechol-functionalized tribenzotriquinacenes, consisting of 89° angle between two catechol units and the 1,4-phenylene diboronic acid leads^143^ (Figure 14b) to the production of the highly symmetrical cubic cage **57**. By varying the choice of diboronic acids with “bite” angles of 60°, 120°, and 180°, a series of boronic ester cages with different shapes can be prescribed, and these boronic ester cages self-select specific permutations in the course of condensation with multiple organic linkers.^144^ Likewise, the self-condensation of diboronic acids with C–B bond angles of 60°, 84°, and 117° can afford a series of polyhedral 3-mer, 6-mer, and 12-mer boroxine cages,^145^ which have exceptional shapes and cavities surrounded by two, four, and eight Lewis acidic boroxines.

In contrast with imine-linked cages, ultralarge shape-persistent boronic cages remain elusive, since the boronic ester bonds are prone to dissociation under acidic, alkaline, and high-humidity conditions. Nonetheless, a significant breakthrough in this field occurred (Figure 14c) with the construction of a giant boronic ester cage (**58**) in 70% yield^146^ as the result of a [8 + 12] condensation of 1,3,5-benzenetri(boronic acid) and a tetraol. The cage was modified by an alkene metathesis of its 48 peripheral terminal alkene units to give a stable exoskeleton, resulting in cage **59** with a large outer diameter of 3.75 nm.

#### 3.2.3 Alkene/Alkyne Metathesis

In dynamic covalent chemistry (DCC), the metathesis of alkenes and alkynes is an emerging strategy for the construction of organic molecular cages. Metathesis refers to the cleavage of double or triple bonds first of all and then recombination to form new alkenes or alkynes as a result of catalytic activity presided over by transition metals. In 2003, porphyrin-based molecular cages were self-assembled^147^ from six Zn(II) porphyrins by alkene metathesis using pyridine-containing thiol-functionalized gold clusters as templates. Subsequently, a four-linked cofacial porphyrinic cage was obtained^148^ in 40% yield by using 1,4-diazabicyclo[2.2.2]octane as a template in the metathesis of porphyrin derivatives containing terminal alkenes. Such cages feature sandwich-type structures, in which 1,4-diazabicyclo[2.2.2]octane molecules are coordinated with the upper and lower porphyrin layers through Zn–N bonds, as confirmed by single-crystal X-ray diffraction analysis. The template and Zn atoms can be removed under acidic conditions to produce a molecular cage with a ligand-free cavity and flexible conformation. Compared with alkene metathesis, the cages constructed by alkyne metathesis have been found to be more stable. In 2011, a rectangular prism porphyrin cage was obtained^149^ by alkyne metathesis without the use of a template. Thereafter, a permanently interlocked organic cage^150^ was prepared by alkyne metathesis, and a tetrahedral organic cage^151^ was synthesized by triple bond-containing precursors employing alkyne metathesis with a yield of 99%.

#### 3.2.4 Other Examples of Dynamic Covalent Chemistry

There are, in principle, other types of reversible bond formations that can be used for constructing organic molecular cages. Labile disulfide (−S–S−) bonds, for example, can be used for constructing shape-persistent cages. In an early example, a trithiol-containing bowl-shaped compound was self-assembled into an organic cage^152^ in the presence of O_2_ or I_2_ with yields as high as 90% by forming reversible disulfide bonds. Hydrophobic factors and the absence of a metal template afforded a trefoil knot^153^ from dithiol-based organic linkers by a slow oxidation process with a yield of 94% simply by increasing the concentration of organic linkers on the addition of NaNO_3._ Furthermore, water-soluble interlocked [3]catenanes were obtained^154^ in a one-pot reaction of the hydrophobic and electron-deficient 1,4,5,8-naphthalenediamide linker acceptor and the electron-rich 2,6-dialkoxynaphthalene donor with the addition of spermine as a template. Compared to the one-pot reaction, the yields were raised to 60% by the stepwise addition of the donor and spermine. In addition to disulfide bond formation, the condensation of a hydrazide with an aldehyde can also be applied to form organic molecular cages. In one report, the dehydration-by-condensation of a formyl and hydrazine derivative (amino) in acidic aqueous conditions led^155^ to the formation of a mechanically interlocked 3D catenane cage. These interlocked cages were decomposed into two discrete cages with the addition of dimethyl sulfoxide.

**Figure 15:**
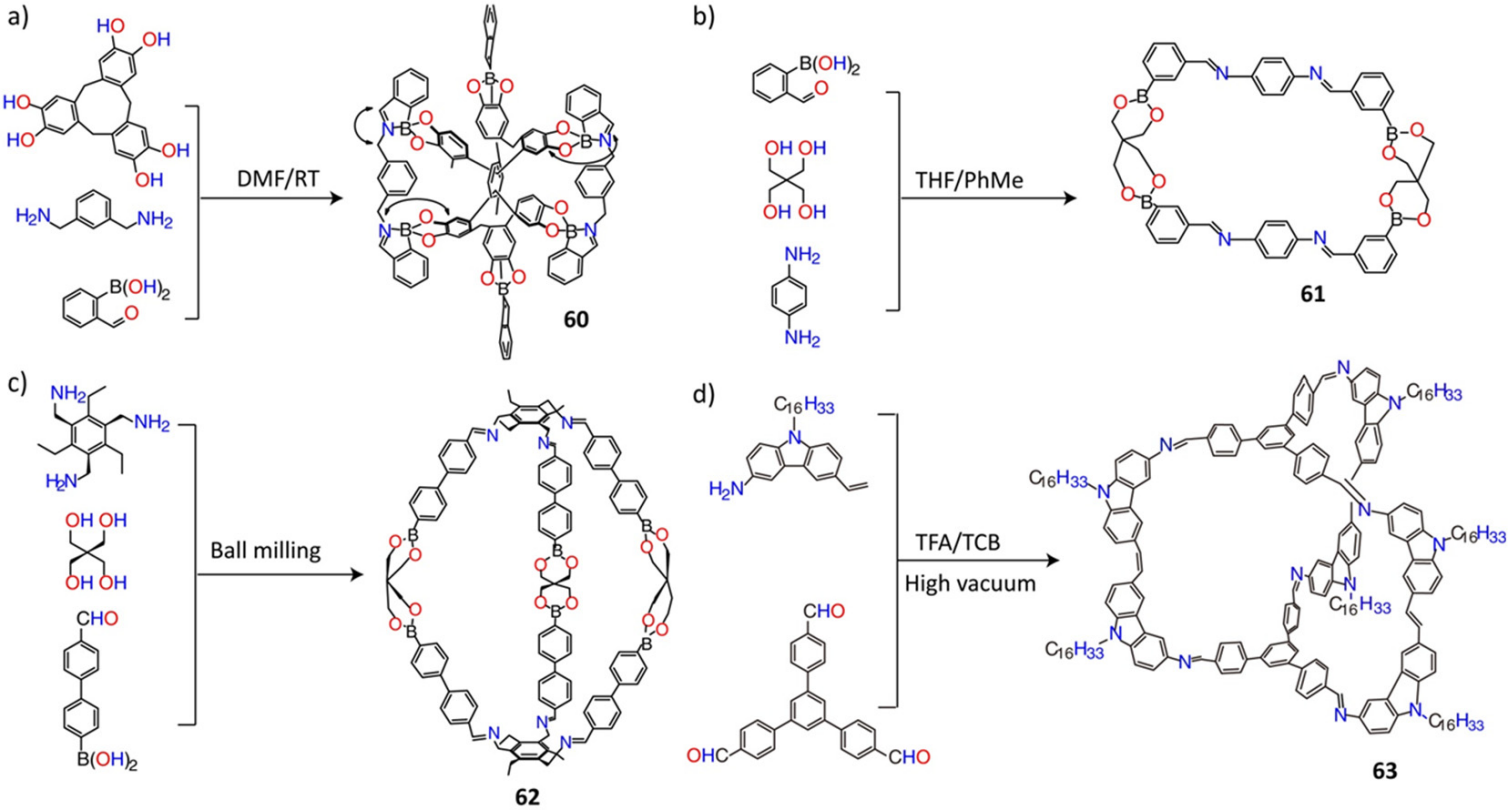
(a) Synthesis of molecular cage **60**. Reproduced with permission from ref (156). Copyright 2008 Wiley-VCH. (b) Synthesis of macrocycle **61**. Reproduced with permission from ref (157). Copyright 2008 Wiley-VCH. (c) Synthesis of molecular cage **62** by ball milling. Reproduced with permission from ref (158). Copyright 2009 American Chemical Society. (d) Assembly of molecular cage **63** by the combination of imine condensation and alkene metathesis. TCB: 1,2,4-trichlorobenzene. Reproduced with permission from ref (161). Copyright 2013 American Chemical Society.

For more complex structures, two different types of reversible bond formation can be used in a synchronous manner to construct POCs. A preferred combination is dynamic imine and boronic ester condensations, because the Lewis acidic boronic acid catalyzes imine condensation. Notably, in some cases, the boronic acid can also catalyze imine-bond hydrolysis, adding another level of reversibility to the reaction. Mixing cyclotricatechylene containing six phenolic hydroxyl groups, *m*-xylylenediamine, and 2-formylphenylboronic acid in deuterated DMF at room temperature leads^156^ to the formation (Figure 15a) of the trigonal cage **60**, while a mixture of 1,4-diaminobenzene, 3-formylphenylboronic acid, and pentaerythritol in THF/toluene leads^157^ to the formation of the macrocycle **61** in 44% yield (Figure 15b) along with insoluble polymeric side material. Solvent-free synthesis has been found to promote the combination of imine and boronic ester condensation. In one report,^158^ the ball milling of pentaerythritol, 4-(4-formylphenyl)phenylboronic acid, and 1,3,5-trisaminomethyl-2,4,6-triethylbenzene at 20 Hz for 1 h produced (Figure 15c) a large [6 + 3 + 2] cage **62** with a size of up to 3.1 nm and a yield of 71%. The same cage was not obtained in solution. The ball milling of *t*Bu_2_Si(OH)_2_, 4-formylbenzeneboronic acid, and 4,4′-bis(aminomethyl)biphenyl has led^159^ to the production of a borasiloxane macrocycle in 85% yield, while the same macrocycle was obtained at a much lower yield (20%) in solution.

The boronic ester functionality has a high affinity for coordination with N-ligands such as pyridines. Therefore, its combination with B–N bonds is promising when it comes to the construction of novel organic molecular cages. An example is the formation^160^ of a cage as a result of the reaction of 2,4,6-tri(4-pyridyl)-1,3,5-triazine, 1,4-benzenediboronic acid, and 4,5-dichlorocatechol. The dative B–N interactions function as a stabilizer for the cage structure. Further, the combination^161^ of imine condensation and alkene metathesis has also been explored. The cage **63** was obtained (Figure 15d) in 51% yield by a one-pot reaction of large-sized carbazole derivatives and trialdehyde compounds.

## 4 Characterization of Porous Organic Cages

Advanced analytical techniques have a critical role to play in the fast-growing field of POCs. The key information about POCs, such as constitutions, structures, and physicochemical properties, can all be revealed comprehensively through an assortment of analytical tools. In this section, we will describe multiple advanced analytical techniques that can offer near-complete characterization of POCs, and thus pave the way for their applications.

**Figure 16:**
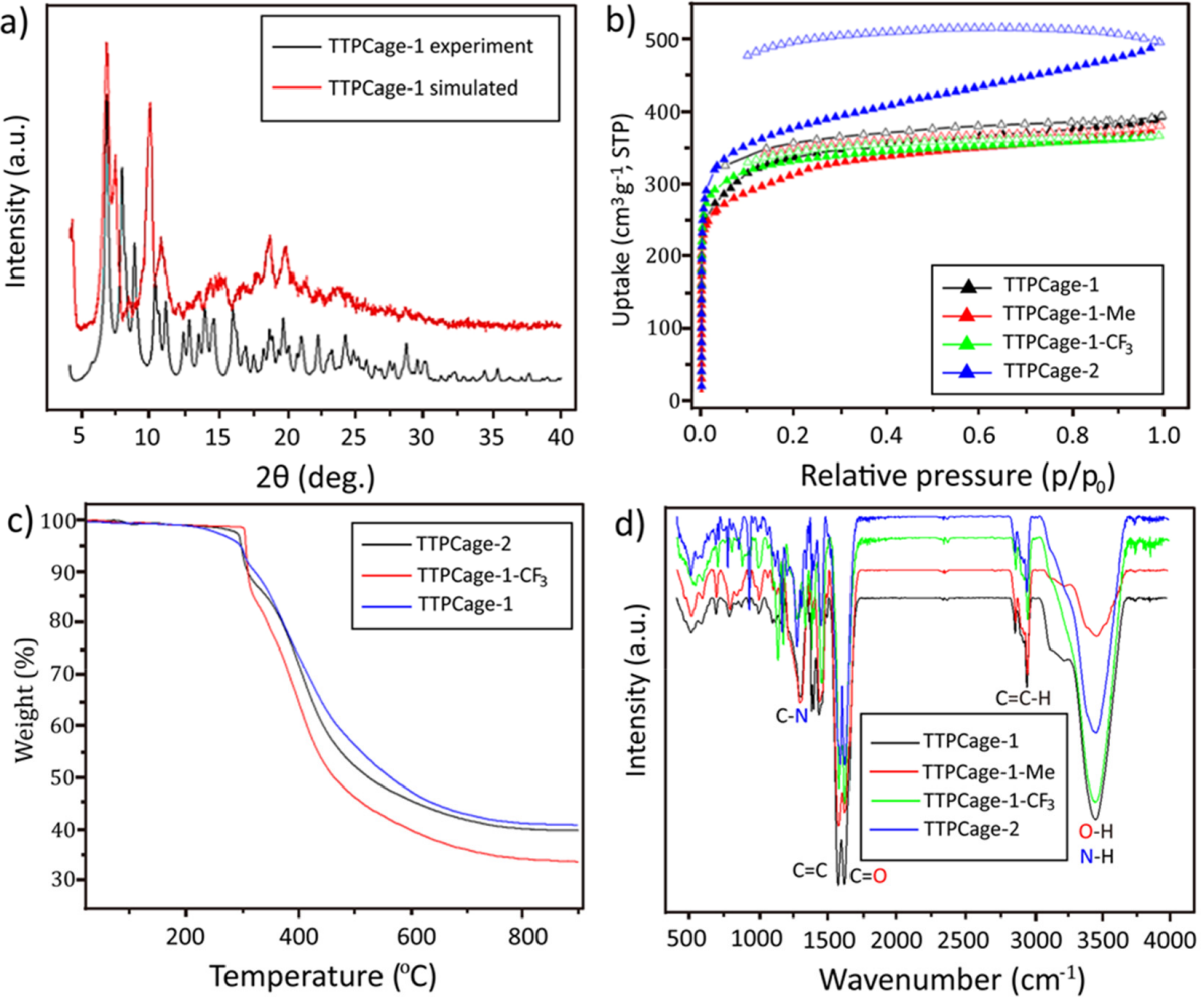
Frequently used analytical tools for investigating POCs. (a) PXRD pattern of the trimeric triangular prism cage (**TTPCage-1**) after desolvation. (b) N_2_ gas sorption isotherms at 77 K for a series of [3 + 6] TTP cages. (c) TGA curves of a series of [3 + 6] TTP cages. (d) FTIR spectra of a series of [3 + 6] TTP cages. Reproduced with permission from ref (127). Copyright 2020 American Chemical Society.

As the most conclusive method (Figure 16a) of analysis, single-crystal/powder X-ray diffraction (SC/PXRD) measurements are key to determine the phase, crystallinity, purity, structural integrity, and crystal size of POCs. SCXRD can reveal the complex structure, noncovalent bonding interactions, and crystal size of a cage structure, provided the growth of a sufficiently large single crystal is possible.^119,120,127^ For example, the truncated cuboctahedron structure of cage **36** has been revealed by using SCXRD.^119^ Four porphyrins in cage **36a** were linked by anticonformation of imine bonds, arranged along the mirror plane and perpendicular to the central 4-fold axis, while the residual eight porphyrins were linked by mixed conformation of imine bonds, and located on both sides of the said mirror plane. Thus, cage **36** has exhibited a large outer diameter of 5.3 nm (maximum ∼7.0 nm, including alkyl chains), with an inner diameter around 4.3 nm. In another example,^120^ by using SCXRD data, the chiral three-bladed propeller structure of cage **37** was confirmed. Cage **37** crystallized in the orthorhombic space group, *Pca*21, with 2-fold and 4-fold rotational symmetries. In addition, some cage compounds are not as robust and their microstructures are prone to collapse after the removal of guest solvents. This behavior holds true even for some resilient cages, where structures are found to decompose under harsh conditions. In this regard, SC/PXRD can also provide direct evidence of cages which have changed their phase, structure or become amorphous after desolvation or other treatments. For example, close inspection of the XRD data revealed that^64^ cage **5** has a flexible molecular structure after the removal of dichloromethane inside its cavities, and its collapsed structure would be recovered to the original crystalline state when a large amount of dichloromethane was adsorbed within its cavities.

The porosity and specific surface areas are important features for POCs. Consequently, N_2_ sorption has become the common tool (Figure 16b) for the determination of their pore size, pore volume, and specific surface area.^122,127^ The gas sorption behavior of POCs is closely associated with their activation methods. For example, as confirmed by N_2_ sorption at 77 K,^122^ the apparent specific surface areas (Brunauer–Emmett–Teller model, BET) of cages **39a** and **39b** activated by thermal treatment under vacuum have never exceeded 11 and 27 m^2^ g^–1^, respectively. Surprisingly, their specific surface areas have been substantially increased to 71 and 443 m^2^ g^–1^, respectively, after room-temperature activation in *n*-pentane with sonication. In addition, N_2_ isotherms have revealed^127^ that the N_2_ capacities and BET surface areas of a series of [3 + 6] triangular prism cages were significantly improved with the increase in their linker length (Figure 16b). Notably, porous structures are very sensitive to N_2_ sorption and changes in gas uptake can be observed immediately by the decomposition of a small percentage of the cage. The PXRD patterns, on the other hand, do not change much with minor incursions. As a consequence, a combination of the SC/PXRD and N_2_ sorption techniques ensures the real-time monitoring of cage phase and structure changes before and after the cage’s use. Thermogravimetric analysis (TGA) can play a complementary role (Figure 16c) to these two key analyses, especially when it comes to determining the thermal stability of cages^127,162^ under varying atmospheric conditions.

POCs have a clear advantage over other extended porous frameworks, i.e., MOFs, COFs, etc., since modern analytical techniques that are available for small molecules can also be employed routinely for the characterization of POCs, owing to their solution processability. High-performance liquid chromatography (HPLC) has been used^163^ for the determination of the optimized reaction conditions for growing crystalline cages, as well as for the purification of the cages. High-resolution ^1^H and ^13^C nuclear magnetic resonance (NMR) spectroscopic analyses are often used^68,162,164^ to reveal the chemical coordination environment, nature of assembly (discrete or otherwise), and symmetry (or lack thereof) of a cage. In one example,^68^ by real-time detection of C5-proton shifts in the triazolium ring through time-dependent ^1^H NMR spectra, the acceleration in the crystallization of cage **8** by the catalysis of 1,2,4-triazolium poly(ionic liquid)s has been confirmed. In another example,^119^ by using ^1^H NMR spectra, the absence of any free aldehyde group in the symmetric structure of cage **36b** has been confirmed. A close inspection of the deshielding in the chemical shift of the hydroxyl protons revealed the formation of intramolecular hydrogen bonds in cage **36b**. In order to characterize POCs on the basis of their size, shape, and association between the cage (host) and nanoparticles (guest), two-dimensional diffusion ordered spectroscopy (DOSY) NMR is remarkably well suited.^119,163,164^ The occasional overlapping of the signals, however, can occur in ^1^H NMR spectra if impure or low-symmetry POCs are present. In order to address the problem, high-resolution electrospray ionization mass spectrometry (ESI-TOF-MS) analysis provides the ideal choice, since it produces distinct patterns with which to analyze the finite structures of POCs.^127,161,162^ Furthermore, dynamic light scattering (DLS) measurements can also be used to distinguish cages on the basis of their grain and pore sizes.^163,164^ For example, the measurement of cage particle sizes by DLS has confirmed^164^ the formation of core–shell structures.

Microstructural and morphological analyses of POCs and POC composites are of considerable value for the cross-validation of textural properties. Scanning electron microscopy (SEM) is the most effective way to visualize directly the size and morphology of POCs on a large scale.^164^ In addition, transmission electron microscopy (TEM), high-resolution TEM (HR-TEM), and high-angle annular dark-field scanning TEM (HAADF-STEM) are often used to visualize the size, distribution, and morphology of single cages or their composites.^163−165^ For example, by using SEM and TEM measurements,^165^ the polyhedral colloidal morphology with mesopores within colloids has been observed for cage **8** synthesized under the long chain ionic surfactant-containing reaction solution. In addition to these electron microscopic techniques, Fourier transform infrared spectroscopy (FTIR, Figure 16d),^127^ X-ray photoelectron spectroscopy (XPS),^164^ and UV–vis absorption spectra^165^ have all been used to investigate the functional groups, binding energies, and photoelectronic properties of POCs, since they are very sensitive to oxidation states and coordination number of the guests.

## 5 Applications of Porous Organic Cages

Research over the past two decades has produced a number of POCs with desirable sizes, porosities, specific surface areas, geometries, and solubilities thanks to both irreversible linking chemistry and DCC. With the help of advanced analytical techniques, their unique physicochemical properties have been well characterized and evaluated, allowing the potential for applications to expand rapidly. Here, we outline and discuss the up-and-coming applications of the POCs, including for molecular recognition, gas storage and separation, porous liquids, porous membranes, heterogeneous catalysis, and as proton conducting materials, in the modern era of chemistry and materials science.

### 5.1 Molecular Recognition and Sensing

Molecular recognition is widely used in nature to regulate biological processes.^166^ A fundamental aim of supramolecular chemistry is to construct novel receptors^167^ endowed with high selectivities, good binding affinities, and unique functionalities toward target molecules, akin to bioreceptors. In this respect, POCs are ideal artificial receptors for guest molecules,^168^ because of their well-defined sizes, inherent 3D cavities, isolated molecular structures, and rich functionalities. More specifically, in many cases, POCs can offer outstanding complementarity to the target guests, even under external, competing stimuli.

POCs are reported to be highly selective receptors for a number of cations, such as Fe^3+^, Ni^2+^, and Ag^+^, and anions,^47,85,99,169^ such as SO_4_^2–^ and Cl^–^. Cylindrical imine cages,^170^ in particular, show high binding affinity for alkali metal cations depending on the nature of their cavities. Small cations, such as Li^+^, Na^+^, K^+^, prefer to bind at the outer surface of the cages, and large cations, such as Rb^+^ and Cs^+^, prefer to reside inside the cavities of the cages. A tetracationic cyclophane, for example, has been found to exhibit^110^ reversible allosteric control of ferrocene under the utilization of PdCl_2_ as a heterotropic effector. The binding affinity of ferrocene can be enhanced or diminished by the stepwise addition or removal of PdCl_2_, respectively. In the case of another example, aromatic oligoamide macrocycles^93^ exhibit outstanding selectivity when it comes to recognizing guanidinium ions.

POCs are also ideal artificial receptors for biomolecules. For example, the recognition of carbohydrates in hydroxyl-rich media is very challenging on account of their complex structures and the lack (generally speaking) of distinct characteristics such as ionic or strongly hydrophobic groups. As a result of the formation of effective intermolecular hydrogen bonds and [CH···π] interactions, the tricyclic polyamide cage **18** (Figure 6a) with two biphenyl and eight amide groups shows^88^ excellent affinity and selectivity for carbohydrates in chloroform, which is maintained in the presence of 8% CD_3_OH. Furthermore, by enhancing the lipophilicity of these polyamide cages by incorporating benzyl substituents, the cages exhibit much higher affinities and selectivities for extracting monosaccharides from water into chloroform.^89^ Another example is porphyrins, which are important in many biological processes, such as oxygen transport, photosynthesis, and metabolism. The X-shaped octacationic cyclophane cages,^111^ which have large and rigid binding cavities, serve as excellent receptors for both free-base and zinc-porphyrins with subnanomolar affinities in water. These high affinities can be attributed to the hydrophobic effect and multiple [CH···π] interactions between the cages and porphyrins. These cages modulate the physical properties and chemical reactivities of the encapsulated porphyrins.

The identification of small harmful molecules, such as toxins in blood and wastewater, is crucial for human health. In this respect, organic cages,^171^ synthesized from the condensation of 4,4′-diformyltriphenylamine and triamines, followed by sodium borohydride reduction, have been utilized as fluorescent sensors for picric acid, a common constituent in many dyes. The high selectivity of these cages for picric acid can be attributed to the formation of a strong cage-picrate complex according to the transfer of the acidic hydroxyl protons of picric acid to basic amine groups in the cages. Moreover, the hexacationic triangular prismatic cages,^113^ which have two 2,4,6-triphenyl-1,3,5-triazine platforms connected by three 4,4′-bipyridinium pillar-shaped spacers, exhibit excellent recognition for polycyclic aromatic hydrocarbons, including pyrene and pyrene-1-carbaldehyde. More specifically, owing to the dipole–cation and dipole–dipole interactions, pyrene-1-carbaldehyde displays a significantly enhanced affinity for binding inside the cage cavity when compared to pyrene, which is usually considered to be the better π-electron donor.

**Figure 17:**
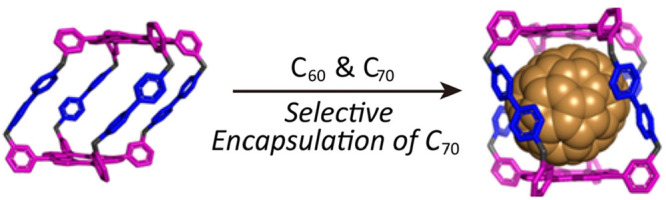
Encapsulation of C_60_ and C_70_ by the cationic molecular cage **TPPCage**^8+^. Reproduced with permission from ref (114). Copyright 2018 American Chemical Society.

Fullerene receptors based on noncovalent bonding chemistry^172^ remain a focus of intense research in supramolecular chemistry. POCs with suitable pore sizes exhibit excellent selectivities and binding affinities for fullerenes on account of their geometric match. For example, C_60_ and C_70_ are difficult to separate because of their structural similarity and nearly identical physical and chemical properties. Zhang and co-workers^149^ have synthesized a rigid porphyrin-based cage, which exhibits a high selectivity of binding of C_70_ over C_60_. The change in distance between the cage and the fullerene guest amounts to a large difference in the stabilization energy. We have reported the synthesis of a cationic molecular cage consisting of two tetraphenyl porphyrins bridged face-to-face by four viologen units,^114^ which are capable of encapsulating both C_60_ and C_70_ courtesy of [π···π], [C–H···π], and [cation···π] interactions. The cage, which shows (Figure 17) a much higher binding selectivity for the larger, ellipsoidal C_70_ over the icosahedral C_60_, results in a selective extraction of C_70_ from a C_60_-enriched fullerene mixture.

In a recent investigation, electrons have been employed^173^ as catalysts for molecular recognition. The formation of a trisradical complex^174^ by a macrocyclic host and a dumbbell-shaped guest is found to be driven by the dynamic addition and extraction of electrons through means of molecular or electrolytic electron donation, enabling a molecular recognition process, that is otherwise kinetically unfavorable because of the steep activation energy. The discovery of a chemical electron source as a catalyst,^173^ has far reaching consequences, for example, POCs with selective guest uptake abilities could now be constructed.

### 5.2 Gas Storage and Separation

Since global warming is attributed commonly to carbon emissions, gas capture, separation, and storage are closely related to the well-being of human society. POCs have received significant attention ever since their gas adsorption capability^50^ was first demonstrated in 2009. The persistent porosity and apparent BET surface areas of POCs and other porous solids, such as zeolites, activated carbons, porous polymers, MOFs, and COFs, are often evaluated by N_2_ adsorption isotherms.^19^ The current record for BET surface area for a POC is reported^140^ to be 3,758 m^2^ g^–1^. The high surface areas lead to potential applications in (i) the adsorption of hydrogen, greenhouse gases, and hydrocarbons, (ii) the removal of toxic gases, and (iii) the separation of light gas pairs (e.g., N_2_/O_2_) for industrial applications.

Hydrogen is an ideal energy source^175,176^ for a sustainable future because of its high energy density and clean combustion. Considering their large surface area, high porosity and tunable structures, POCs have considerable potential for hydrogen storage. In 2009, a shape-persistent POC (**10**, Figure 4) was reported.^50^ The molecular structure of **10** includes six vertex methyl groups and packs to form a microporous solid with a window-to-arene arrangement and a 1D pore channel. This cage is on record for taking up 8.88 mmol g^–1^ H_2_ (1.75 wt %) at 77.3 K and 7.0 bar. Furthermore, a soft POC, **9**, has been reported,^177^ and, after desolvation, was found to adsorb 9.49 mmol g^–1^ H_2_ at 77.3 K and 1.2 bar. Another organic cage **7**, prepared^50^ in ethyl acetate, features four arene faces and four triangular windows and packs to form a nonporous solid. The desolvation of **7**/ethyl acetate by heating leads to the formation of a polymorph, **7**α′, which is nonporous to N_2_ and H_2_ at 77 K. The exposure of **7**α′ to dichloromethane vapor, followed by complete desolvation under vacuum at 383 K, led to the formation^78^ of a new polymorph, **7**β′, which is nonporous to both N_2_ and Ar at 77 K and yet adsorbs significant quantities of H_2_. In addition, by making asymmetric organic cages through dynamic covalent scrambling reactions, amorphous cage solids have been formed.^178^ These cages boast enhanced H_2_ storage properties compared to their crystalline counterparts. Notably, despite the progress during the past few years, improvement in the H_2_ storage capability of POCs is still one of the most important research directions that needs to be addressed with same intensity in the future.

In the capture and storage of the greenhouse gases CO_2_ and CH_4_, POCs can play an important role on account of their tunable adsorptive sites in addition to their large surface areas. A series of imine cages,^50,120,179^ such as **7**–**10** (Figure 4) and **37** (Figure 10), have been developed for adsorbing large quantities of CO_2_ and CH_4_ at high-pressures (140 bar). Likewise, porous tricyclooxacalixarene cages^180^ exhibited good CO_2_ uptakes of up to 12.5 wt % (2.8 mmol g^–1^) and high selectivity for CO_2_ over N_2_ adsorption (CO_2_/N_2_ = 80/1, v/v) at 273 K and 1 bar. Amide-based cages^60^ have also shown enhanced adsorption of CO_2_ and CH_4_, while defective imine cages^181^ were especially notable with their enhanced CO_2_ uptake because of the presence of additional functional groups.

The transformation of POCs into frameworks, while maintaining their structures and inherent cavities, can improve their CO_2_ adsorption capability significantly. In 2011, a 3D-cage framework was constructed^182^ by linking covalently prefabricated imine cages that exhibit high selectivity for the adsorption of CO_2_ (CO_2_/N_2_ = 138/1, v/v). The synergistic effects between functionalized amino groups (from the reduction of imines) and the intrinsic pore size of the cage structure are believed to be responsible for the high CO_2_/N_2_ selectivity. In 2017, a 3D coordination-networked cage was prepared^183^ by using the imine-linked cage as the precursor. This cage showed an enhanced CO_2_ uptake (1093 cm^3^ g^–1^ at 23 bar and 273 K) with a lower CO_2_ adsorption enthalpy than that of the cage precursor. More recently, a cage-based 3D COF was constructed^184^ by applying an organic cage as a triangular prism linker. This COF adsorbed 204 mg g^–1^ CO_2_ at 273 K and 1 bar, and 107 mg g^–1^ at 298 K and 1 bar, one of the highest CO_2_ uptakes for COFs under the same conditions.

**Figure 18:**
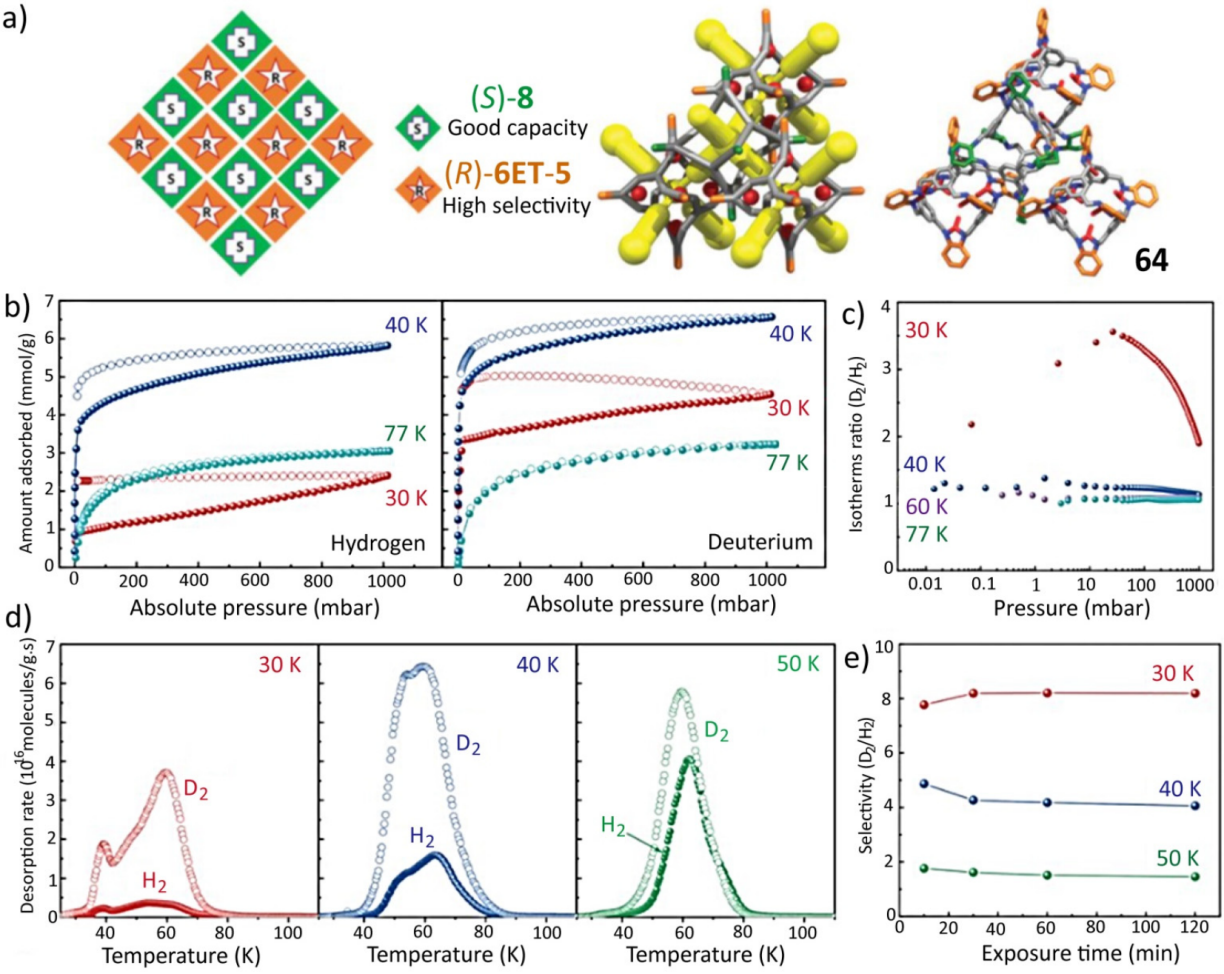
(a) Formation of cocrystal **64** by chiral recognition. (b) H_2_ and D_2_ adsorption (closed symbols) and desorption (open symbols) isotherms of the cocrystal at different temperatures. (c) D_2_/H_2_ Isotherm ratio as a function of pressure at different temperatures. (d) Thermal desorption spectroscopy (TDS) recorded on cocrystal **64**, obtained after exposure to a 10-mbar 1:1 H_2_/D_2_ isotope mixture under varying temperatures (*T*_exp_) for a fixed exposure time (*t*_exp_) of 30 min. (e) D_2_/H_2_ Selectivity as a function of *t*_exp_ at 30 K (red), 40 K (blue), and 50 K (green). Reproduced with permission from ref (186). Copyright 2019 American Association for the Advancement of Science.

POCs are also promising when it comes to the separation of rare gases that are required in high purities for industrial applications. Although the static pore size of the imine cage **8** (Figure 4) is smaller than that of the dynamic radius of Xe, it has been found that^185^ this cage can separate Xe selectively at low concentrations directly from air. The excellent Xe separation performance can be attributed to the near-perfect size match between the cavity of **8** and Xe atoms. The homochiral cage **8** also allows the separation^183^ of a chiral alcohol, 1-phenylethanol, with selectivity for the enantiomer having inverse optical polarization to that of the cage. More recently, a “barely porous” cage (**6ET-5-R**) was synthesized^186^ by the postmodification (Figure 18a) of the reduced imine cage **5**. In combination with the nonporous POC (*R*)-**6ET-5** and the large-pore imine cage **8**, a novel cocrystalline cage **64** was formed, showing (Figure 18b–e) superior selectivity and uptake of the hydrogen isotope, deuterium.

As far as the capture of other industrially prominent gases is concerned, sulfur hexafluoride (SF_6_) is a leading candidate on account of the fact that it is a serious threat to the planet,^187^ with its greenhouse effect being around 24 times higher than that of CO_2_. The imine cage, **8**, exhibits^188^ excellent selective adsorption for SF_6_ over N_2_ (SF_6_:N_2_ = 178/1, 273 K 1 bar; 74/1, 298 K, 1 bar), surpassing most of the reported porous MOFs, such as UiO-66-Zr and Zn-MOF-74. This same cage is also able to separate mesitylene from its structural isomer, 4-ethyltoluene, in addition to separating *m*-xylene from *p*-xylene.^189^ Direct separation was also demonstrated^190−193^ by the deposition of soluble POCs on gas chromatography (GC) columns. Cage-coated GC columns exhibit extremely high selectivity for the separation of a series of constitutional isomers and organic molecules, such as *n*-alkanes, *n*-alcohols, and aromatic hydrocarbons, with enhanced separation in the case of chiral molecules. In addition, both imine-linked cage^194^**8** and per-ethylated pillar[6]arene^195^ showed excellent performance in removing iodine.

### 5.3 Porous Membranes

On account of their low-cost, energy-saving, and high reliability, membrane separation techniques have been proposed alternatives^196^ to conventional thermal separation processes. The key component in membrane separation technology is the filler or matrix materials, which determine the cost, operational reliability, and separation performance. Numerous porous inorganic/organic materials, such as zeolites,^197^ silica,^198^ carbons,^199^ MOFs,^200^ COFs,^201^ and polymers,^202,203^ have been reported for the production of high-performance porous membranes. Most of these porous materials, however, are insoluble in common solvents that can dissolve membrane polymers during their preparation, leading to the uncontrollable distribution of filler materials or unavoidable defects in the resulting membranes. POCs can also serve as ideal fillers or matrix materials in membranes since they exhibit excellent solubility, processability, and porosity. In the event, the POCs offer defect-free porous membranes with homogeneous filler dispersion and good filler–polymer matrix compatibility.

**Figure 19:**
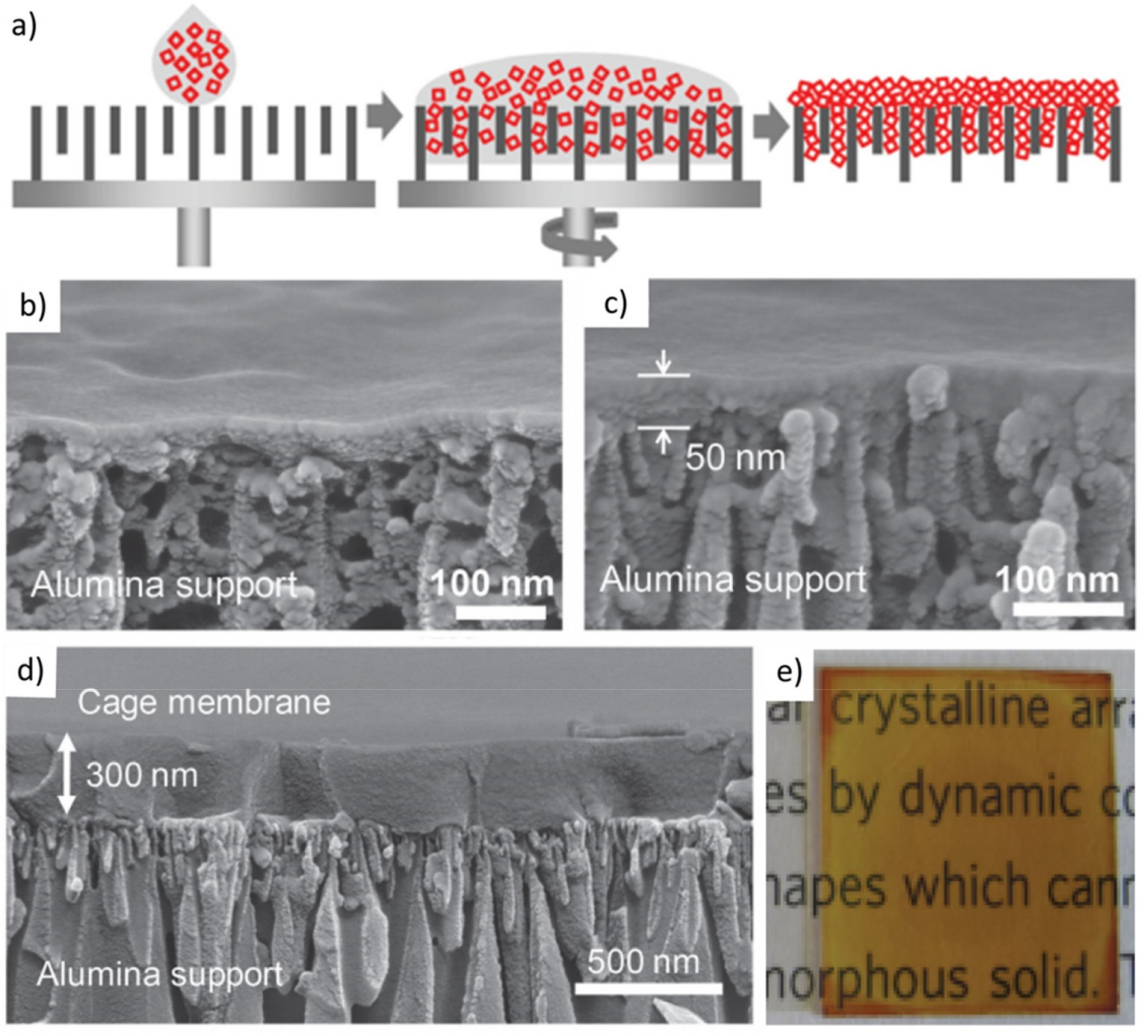
(a) Spin-coating of a porous cage solution to afford an ultrathin cage-film layer on a porous substrate. (b) A cross-sectional SEM image of amorphous cages coated on Al_2_O_3_ support. (c) A cross-sectional SEM image of a 50 nm-thick cage **8** thin film coated on Al_2_O_3_ support. (d) A cross-sectional SEM image of a 300 nm-thick thin film of **8** coated on an alumina support. (e) Photographs of cage thin films spin-coated on glass slides. Reproduced with permission from ref (211). Copyright 2016 Wiley-VCH.

The field of cage-bearing porous membranes is still undergoing development. Recently, computational methods, including Voronoi network analysis,^204,205^ grand canonical Monte Carlo simulations,^205^ and molecular dynamic simulations,^205−208^ have been used to investigate the benefits of using POCs as filler materials for porous membranes. The results reveal that porous membranes containing POCs can exhibit enhanced selectivity and permeability for gas separations and water desalination, when compared with the corresponding neat polymer matrices. Inspired by these computational investigations, experiments have been carried out to realize cage-containing membranes. In 2012, films of 10–30 molecular layers using seven derivatives of adamantanoid triptycene-based salicylbisimine cages were deposited^209^ on a quartz crystal microbalance, representing the first experimental foray into this emerging field. The microporosity of the cage films has been confirmed by the uptake and release of aromatic guests. The quartz crystal microbalance^210^ with deposited cage film is able to detect selectively a target molecule, such as the drug γ-butyrolactone. Furthermore, by using a spin-coating method, researchers have fabricated^211^ (Figure 19) transparent thin films of POCs on different supports, such as glass, silicon, and alumina. The film thickness can be controlled by varying the choice of solvent and the concentration of dissolved POCs. These thin-film composite membranes show molecular-sieving properties and good selectivity toward gases. With a simple dip-coating, the oriented two-dimensional cage layers^212^ can also be deposited on silicon wafers and glass supports. Their structural defects can be observed by atomic force microscopy and the defect concentration can be correlated with the crystallization rate. In addition, imine-based cage membranes can be grown on tubular alumina^213^ by a secondary seeded-growth approach. The resulting membranes were as thin as ∼2.5 μm, and exhibited excellent separations for light gases such as He, CO_2_, CH_4_, and Kr from Xe. More recently, continuous composite membranes^214^ were fabricated by coating closed packed and defect-free films of cage **8**α on polyacrylonitrile (PAN). These membranes (**8**α/PAN) exhibited excellent permeance to both polar and nonpolar solvents, such as water (43.01 m^–2^ h^–1^ bar^–1^) and toluene (55.91 m^–2^ h^–1^ bar^–1^). On the other hand, the cage **8**α/PAN membranes could reversibly switch into the less dense crystalline phase, cage **8**γ′/PAN in methanol, which provided effective pore aperture with different selectivities for high water resistance and excellent organic dyes permeation. Consequently, by varying the water/methanol ratio, these cage **8**/PAN membranes have been utilized as smart, responsive graded molecular sieving for separating organic molecules of different sizes.

POCs can also be blended with nanoporous polymers to make mixed matrix membranes. Early examples have featured porous membranes^215,216^ by incorporating porous imine cages into a nanoporous organic polymer matrix, leading to significantly enhanced permeabilities for mixed gases while retaining high selectivities. Furthermore, a waterwheel-shaped POC, called Noria, and its derivative, Noria-CO^*t*^Bu—a Noria derivative containing *t*-butyl ester groups—have been applied^217^ as filler materials in a fluorine-containing polyimide membrane. The Noria-CO^*t*^Bu displayed a higher surface area, larger pores, and better compatibility with a polyimide matrix when compared to Noria itself, achieving homogeneous dispersion of nanoaggregates and fine adhesion in the resulting membranes. Thus, different performances were exhibited by these two membranes—the Noria-containing polyimide membrane gave an enhancement in CO_2_/CH_4_ selectivity with a decrease in the CH_4_ permeability, while the Noria-Co^*t*^Bu-containing polyimide membrane led to higher free volume and gas permeability. Moreover, the vertex-functionalized amorphous scrambled POCs have been exploited^218^ as filler materials in Matrimid and poly(styrene) membranes, respectively. While amorphous organic cages have been dispersed homogeneously throughout the polymer matrix without any phase separation or aggregation, these membranes provide significant enhancements in both permeability and selectivity for gas pairs and organic solvents.

### 5.4 Porous Liquids

Porosity is a distinctive property that is typically associated only with materials in their solid state. James and co-workers^219^ reported in 2007 that porosity can also exist in the liquid state, despite the fact that it is limited to a small transient void within the molecules. In accordance with the empty voids, porous liquids can be categorized^219^ as neat liquids comprising fluid hosts with (i) rigid, intrinsic, and empty cavities (type I), (ii) a mixed liquid consisting of dissolved empty hosts (type II), and (iii) consistently dispersed framework materials (type III) in sterically hindered solvents.

Because of their convenience, efficient use, and free-flowing properties, porous liquids have gained attention recently for industrial applications such as gas sorption. POCs are among the most promising classes of tunable pore generators in liquids, owing to their rigid backbones, accessible internal cavities, and good solubilities. The goal is to use a solvent whose molecular size is large enough so as to not penetrate into their cavities, providing a sustainable void in the liquid form. It turns out POCs as pore generators make type II porous liquids. Hemicarcerands, which are strongly chelating and highly flexible porous molecules and were reported by Cram and co-workers^220^ in 1994, may very well be the first example of porous liquids that illustrate the concept of dissolving POCs in sterically challenging solvents. The cavities of hemicarcerands are large enough to encapsulate small solvent molecules such as dichloromethane and dimethylacet-amide (Me_2_NCOMe), but too small to accept larger solvents like diphenyl ether. As confirmed by ^1^H NMR spectroscopy, Me_2_NCOMe molecules inside the cavities of hemicarcerands can be removed completely by heating their inclusion complexes in diphenyl ether for 5 days at 468 K. Although guest-free hemicarcerands dissolved in diphenyl ether behave like a type II porous liquid, there was no direct evidence at the time to prove that the empty cavities of hemicarcerands were retained after the removal of the guest solvent Me_2_NCOMe. Most recent experiments have been designed to prove porous liquid behavior by dissolving POCs in sterically hindered solvents. Subsequent attempts, however, failed^221,222^ to prove porosity since the material introduced could not sustain the free voids for a reasonably long time.

**Figure 20:**
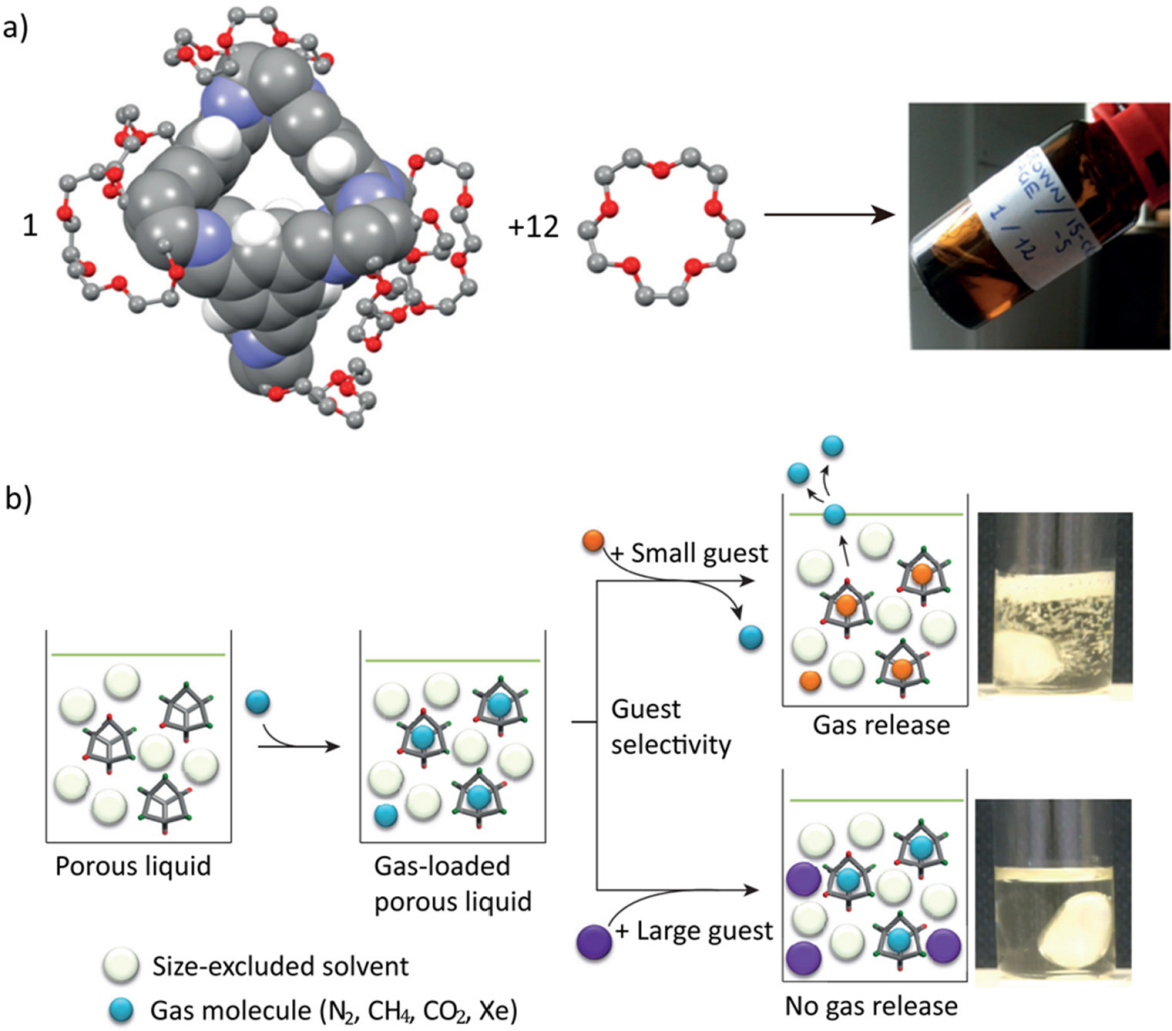
(a) Porous liquids obtained by empty, soluble cage molecules with [15]crown-5 as a solvent. (b) The porous liquids show enhanced solubilities for guest gas molecules. Reproduced with permission from ref (223). Copyright 2015 Springer-Nature.

Twenty years later in 2015, James and co-workers^223^ reported sustained porosity in a type II porous liquid. A tetrahedral POC was functionalized (Figure 20a) with six crown ethers and dissolved at extremely high concentration (44%) in a sterically hindered 15-crown-5 solvent system. The existence of stable voids in porous cages in [15]crown-5 acting as the solvent was confirmed by both molecular dynamic simulations and positron (*e*^+^) annihilation lifetime spectroscopy (PALS) experiments. Furthermore, these authors demonstrated (Figure 20b) that the solubility of guest molecules in such porous liquids can be improved significantly. For example, methane solubility has been increased 8-fold in the porous liquid at 305 K in contrast to the pure [15]crown-5 solvent. In the same investigation,^223^ an easy-to-prepare type II porous liquid was synthesized by dissolving a cage mixture in a hexachloropropene, a solvent which is estimated to be too big to enter the pores of the cage. Since there are ample unoccupied cavities in a cage-hexachloropropene solution, the porous liquids show enhanced solubilities for methane, nitrogen, carbon dioxide, and xenon, compared to that of the nonporous pure hexachloropropene solvent.

The thermodynamics and kinetics of gas storage in crown-ether-cage porous liquids have been investigated^224^ by molecular dynamic simulations. The ability of gas capturing depends primarily on the size/shape of the gas molecules and the noncovalent bonding interactions of the corresponding cage with the gas molecules. The former determines the effective use of the cage cavities, while the latter controls the affinity of gas molecules for the cage. Following this concept, the order of gas storage capacity in POCs has been determined to be CH_4_ > CO_2_ > N_2_. In 2017, Greenaway et al.^225^ explored the development of vertex-disordered porous liquids using a scrambled-cage approach. As a result of studying a total of 150 combinations of different bulky solvents and scrambled cages, it has been suggested that the cage cavities remain empty even in the absence of a suitable guest, and that the liquids adsorb reversibly large quantities of gas, e.g., 72% for Xe and 74% for SF_6_. The authors were of the belief that some physical properties of cage solids were translated into the porous liquid, while retaining their gas binding affinity. This explanation, however, was not found to be the valid for every cage. A solid homochiral cage showed enantioselectivity for chiral aromatic alcohols, whereas the corresponding porous liquid exhibited no such selectivity.^226^ Consequently, methods have been developed to translate the properties of porous cages into porous liquids. The size restraint from the pore opening is considered a standard approach for tuning the gas selectivity in porous solids. Recently, it was reported^226^ that a POC-based, type II porous liquid can switch its selectivity from Xe to CH_4_ simply by reducing the cage pore size.

Type I porous liquids, on the other hand, can be formed by the direct liquefication of POCs. James and co-workers^221,222^ found that, if porous imine cages are functionalized with long alkyl chains, they can form neat liquids with low melting points. The tails of the long alkyl chains, however, have been found to occupy the cage cavities, removing the porosity. Surprisingly, in a more recent study,^227^ a type I porous liquid has been developed by simply mixing [18]crown-6 as the solvent with an anionic POC. In the resulting charge-neutral porous liquid combined, the anionic parts are from the anionic POCs and cationic parts from [18]crown-6/potassium-ion complexes. The presence of persistent free voids in these simply-made porous liquids was demonstrated by molecular dynamics simulations and PALS experiments. Unlike the pure [18]crown-6 solvent, the porous liquids so obtained showed enhanced affinity for CO_2_. It is worth noting that in the preparation of a new generation of porous liquids, POCs and MOFs may complement with each other with their own advantages. POCs are promising for the formation of type I and type II porous liquids owing to their solubility in common solvents. MOFs, on the other hand, feature tunability in pore size and types in their superstructures and their insoluble dispersions^228^ make them important candidates for type III porous liquids. In addition, by functionalizing hollow silica spheres with suitable corona and canopy species,^229^ type I porous liquids containing silica cavities have been synthesized for gas separation. In combination with POCs, these inorganic analogs are also promising for the new generation of porous liquids, as they usually generate more free volumes in the liquid.

### 5.5 Heterogeneous Catalysis

Metal nanoparticles have attracted significant attention in the field of catalysis^163,230−232^ as a consequence of their exceptional catalytic performances and recyclability. The controlled synthesis of metal nanoparticles, however, is very challenging since aggregation of the metal nanoparticles driven by their high surface energy is almost unavoidable. Some POCs can stabilize metal nanoparticles by wrapping themselves around them, on account of their versatile functionality, small-sized voids, large surface areas, high porosities, and, most importantly, their excellent solubilities in common solvents. The strong chelating and stable confinement abilities of the small cavities of POCs can be used to isolate ultrafine, uniformly distributed, and highly stable metal nanoparticles. Moreover, POC-stabilized metal nanoparticles offer unique potential because of their similarities to homogeneous catalysts, while providing heterogeneous catalyst-like features on their surfaces.

Even without the metal clusters in their cavities, POCs lend themselves to use as heterogeneous catalysts because of their diverse functionalities. To the best of our knowledge, only two examples of catalysis by POCs themselves have been reported.^233,234^ A proof-of-concept synergistic catalytic system for the cycloaddition of CO_2_ and propylene oxide has been reported by Patra and co-workers^233^ in which a shape-persistent *N*-rich amine cage, prepared by the reduction of the imine cage **7** (Figure 4), was applied as a catalyst and tetra-*n*-butylammonium bromide was used as the cocatalyst. In another example, a porphyrin-based tubular organic cage has been reported^234^ for the photocatalytic oxidative coupling of amines under visible light. Unfortunately, the overall catalytic activity was quite low, possibly because POCs contain lots of saturated covalent bonds and are devoid of metal centers.

Imine-based POCs feature cage cavities with ample imine/amine functionalities that can capture and confine metal nanoparticles efficiently with ultrafine size and unmatched dispersibility. In this regard, a series of palladium (Pd) nanoparticles of varying sizes (1.0 to 3.0 nm) have been synthesized^235−238^ by solution impregnation, where MeOH, NaBH_4_, or H_2_ was used as a reducing agent. The metal nanoparticles can be (i) anchored on the outer surfaces of imine cages, (ii) positioned on the embedded edges of cage crystals, or (iii) located within the cage cavities. These POC-metal nanoparticle composites display excellent catalytic activities and stabilities for the carbonylation of aryl halides,^235^ CO oxidation,^236^ Tsuji-Trost allylation,^237^ and 4-nitrophenol hydrogenation.^238^ In addition, when well-dispersed Rh nanoparticles with an average diameter of 1.1 nm are loaded on the outer surfaces of the imine cage **8** (Figure 4) by a wet chemical reduction method,^239^ the metal-decorated cage shows excellent catalytic activity for the methanolysis of ammonia-borane and for the hydrogenation of 4-nitrophenol.

In similar fashion, POCs outfitted with thioether,^240^ carbene,^241^ or phosphine^242^ groups can serve as ideal binding centers for metal nanoparticles. For example, uniformly dispersed Pd nanoparticles with an average diameter of 1.8 nm have been incorporated^240^ within the cavities of thioether-functionalized cages, affording high catalytic activity for the Suzuki-Miyaura reaction. Highly dispersed Au nanoparticles (*d* = 1.98 ± 0.3 nm) have been grown^241^ within the cavities of polyimidazolium cages featuring ample interior *N*-heterocyclic carbenes, yielding a combination that showed outstanding activity and superior durability for the hydrogenation of 4-nitroaniline affording 4-phenylenediamine. Well-dispersed Pd nanoparticles (*d* = 1.7 ± 0.3 nm) have been confined within a new phosphine-bearing cage,^242^ revealing superior catalytic activity and stability in a series of aryl halide cross-coupling reactions.

In the context of strong electrostatic attraction and repulsion, charged organic cages also provide ideal platforms for the stabilization of metal nanoparticles. By simple anion exchange, various noble-metal clusters (<1 nm) have been encapsulated within an ionic organic cage,^243^ which displays excellent catalytic activity for the hydrolysis of ammonia-borane to generate hydrogen.

**Figure 21:**
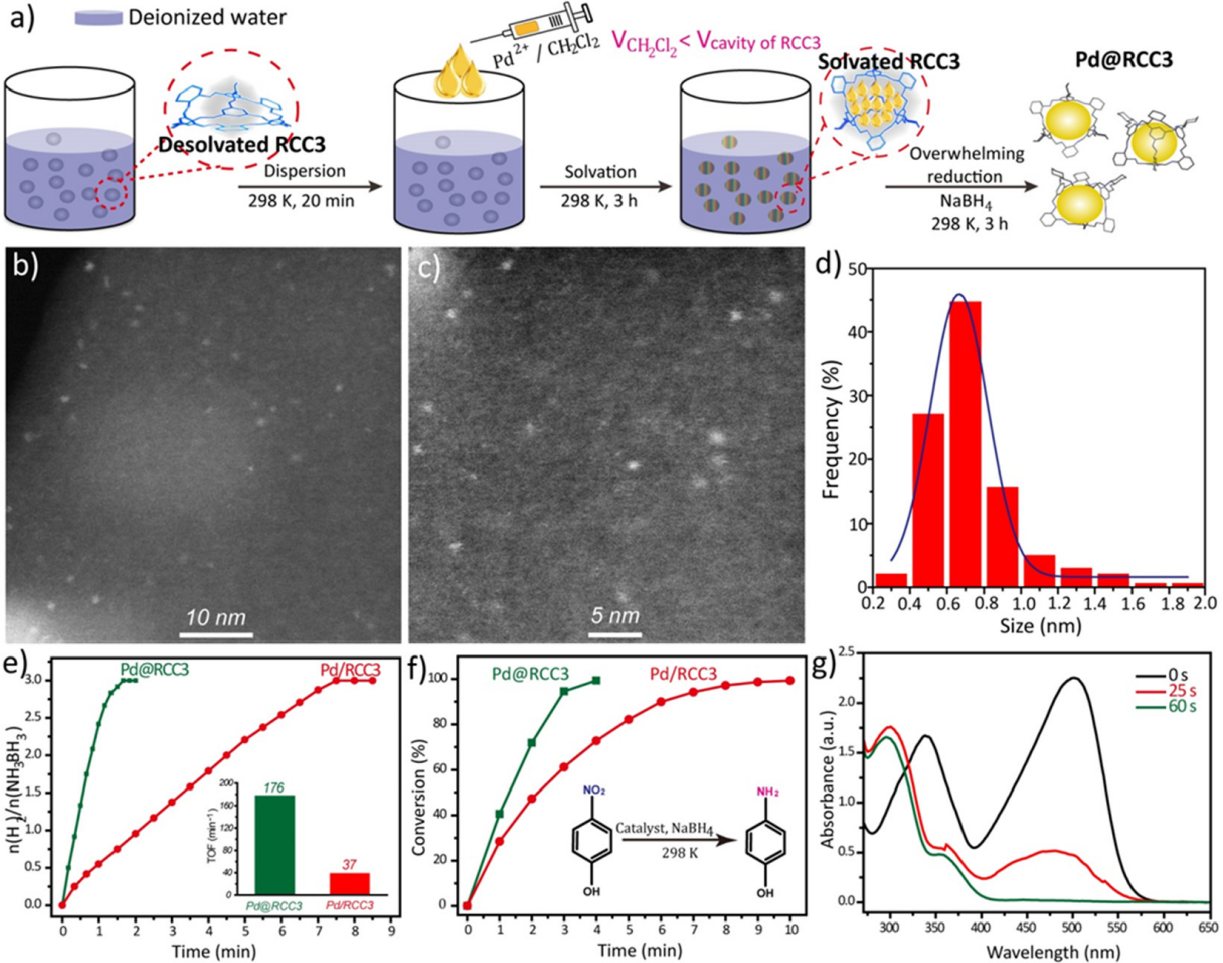
(a) A reverse double-solvent approach has been developed for encapsulating metal clusters inside the cavities of cage **6** (Figure 3). (b, c) HAADF-STEM Images and (d) particle size of the obtained Pd clusters. The Pd@**6** showed excellent catalytic activities for (e) hydrogen generation from ammonia borane, (f) hydrogenation of 4-nitrophenol, and (g) reduction of dyes. Reproduced with permission from ref (244). Copyright 2018 Springer-Nature.

In addition to the individual functional groups on POCs, the hydrophobic nature of cage cavities is also found to be quite useful in phase separating substrates during catalysis. In 2018, Xu and co-workers^244^ reported (Figure 21) a novel reverse double-solvent approach for encapsulating ultrafine (∼0.7 nm), well-dispersed and highly stable Pd clusters inside the reduced imine cage **6** (Figure 3). Surprisingly, the resulting Pd clusters exhibit excellent catalytic activity and selectivity for a series of liquid-phase catalytic reactions, including the methanolysis of ammonia-borane, hydrogenation of nitroarenes, and reduction of organic dyes.

Cage-confined metal nanoparticles have also shown promise in photocatalytic reactions. Recently, by using the encapsulation strategy, highly dispersed Au, Ag, and Pd nanoparticles have been developed for photocatalytic reactions, such as the selective reduction of nitroarenes to azo compounds,^245^ the sequential reactions of aerobic hydroxylation,^246^ and hydride reduction of 4-nitrophenylboronic acid.^247^ Furthermore, by depositing Pd nanoparticles, stabilized by POCs (based on cryptands) onto graphitic carbon nitride (g-C_3_N_4_), a new type of Pd@cryptand/g-C_3_N_4_ photocatalyst has been obtained,^248^ leading to outstanding activity for hydrogen production from water with long-term durability.

Recently, cage-confined metal nanoparticles have been used in electrocatalytic reactions. In one report,^249^ monodispersed ferrihydrite nanoparticles (*d* = 1.9 ± 0.3 nm), encapsulated within organic cages by *in situ* nucleation combined with air oxidation, showed excellent redox activity in MeCN. In another report,^250^ a highly dispersed Ru@cage **6** catalyst, fabricated by the reverse double-solvent approach,^244^ has endowed Li–O_2_ batteries with high specific capacity, high-rate capability, and long-term stability.

### 5.6 Other Applications

The cavities of POCs with precise sizes and shapes can be used as microreactors for chemical reactions. The reactive species can be stabilized for a long time owing to pore confinement. For example, highly strained and reactive Bredt olefins are usually unstable in solution with a lifetime of a few minutes. Warmuth and co-workers^251^ have reported hemicarcerand cages, synthesized through the assembly of a cavitand and propylenediamine in water in the presence of 3-noradamantyldiazirine as a template, followed by the encapsulation of protoadamantene within their cavities by the irradiation of its precursor in the form of a trapped diazirine template. The protoadamantene shows high stability in (CD_3_)_2_SO/CD_3_CN for several days at room temperature as a result of protection by the surrounding cage. In addition, the radical polymerization of styrene has been achieved^252^ within the tetrahedral imine cage (Figure 4) with extrinsic porosity. The polymerization is highly reliant on the crystallinity of the cage **8** and is promoted by the flexible cage-packing structure resulting from the adsorption of styrene monomers.

In common with MOFs and COFs, POCs can be employed as candidates in proton-conducting materials. Liu et al.^253^ have reported the synthesis of ionic cages from the hydration of the reduced imine cage **6** (Figure 3), which has a superior proton conductivity when compared to that of Nafion. Moreover, a novel proton-exchange membrane was designed by implementing solution-blowing of sulfonated poly(ether sulfone) (SPES) nanofibers containing the porous imine cage **8** and subsequent filling by a Nafion solution in the interfiber voids.^254^ With the unique synergistic effect of the cage **8**@SPES nanofibers and Nafion, this membrane exhibited high photon conductivity, high water absorption, high thermal stability, and low MeOH permeability.

POCs have been explored for the efficient transport of lithium ions in batteries. The solid–liquid electrolyte nanocomposites^255^ consisting of a bis(trifluoromethane)sulfonamide lithium salt (LiTFSI)/1,2-dimethoxyethane (DME) electrolyte solution and kinetically trapped tetrahedral cages (Td_A_^256^) have been fabricated for lithium-ion batteries, which displayed an exceptional conductivity of 1 × 10^–3^ S cm^–1^ and a low activation energy of 16 kJ mol^–1^ at room temperature, as well as excellent oxidative stability up to 4.7 V. The POC-based ionic conductor,^257^ Li-RCC1-ClO_4_, has been developed for solid-state lithium batteries. The unique solution-processability advantage of such Li^+^-conducting POCs facilitated the formation of highly efficient ion-conducting networks on cathode surface through recrystallization and growth during a slurry-coating process. As expected, the resulting batteries showed low polarization and good cyclability at room temperature.

In addition to proton and Li^+^ transport, POCs have great potential in water transport. In an early study,^258^ cage **8** exhibited excellent water uptake (up to 20.1 wt %) and high stability in boiling water (>4 h). In 2020, Zhao’s group^259^ have found the fast water permeance and high rejection of small cations/anions of various zero-dimensional POCs with nanopores through experiments and simulations. Pore window size, structural rigidity, hydrophilicity, and ability to form interconnected channel networks were responsible for their water- and ion-transport capability. These POCs have great potential in desalination applications due to their unique solution processability for the preparation of homogeneous, composite membranes.

Based on good biocompatibility and low biotoxicity, POCs have been extended to the biological sciences. Peng and Li’s group^260^ have encapsulated Pt nanoclusters within pores of the soluble protonated cage **5** (Pt-*in*-(HR)CC3), which revealed superior effects in short-term and long-term cancer radiotherapy. In addition, inspired by concept of aggregation-induced emission (AIE), the AIE-active positively charged *R*(+)-TPE-cage has been constructed by acidification of a reduced [6 + 8]-type TPE-cage.^261^ The as-obtained AIE-active *R*(+)-TPE-cage displayed excellent external stimuli (temperature and viscosity) and highly efficient live-cell imaging.

Recently, the design and development of artificial molecular machines^262−264^ to simulate various functions of biomacromolecules in living organisms has become one of the contemporary research themes for biologists, chemists, and materials scientists. For example, mechanically interlocked molecules,^265^ such as catenanes^266^ and rotaxanes,^267^ have been explored extensively as artificial molecular machines on the basis of using energy imparted by external stimuli—such as light, redox potential, and chemical reagents—to maintain their nonequilibrium steady state.^268,269^ By using the encapsulation and postmodification strategies, POCs are destined to have broad applications in providing frameworks for housing artificial molecular machines, considering their unique features, e.g., the discrete molecular structures, large guest-accessible cavities, abundant interconnected pores, tunable inner microenvironments, and excellent solubility in common solvents. Since POCs have not yet been integrated with artificial molecular machines, we will limit this discussion to three groundbreaking examples that may inspire readers. One^270^ is the development of a mechanically interlocked suit[3]ane that consists of a benzotrithiophene derivative with three protruding alkyl chains as the body and a 3-fold symmetric, extended pyridinium-based cage as the suit. When the cage as suit is fitted appropriately around the body, this unstable molecule with three flexible alkyl chains as its protruding limbs shows very high stability in CD_3_CN at 100 °C for 7 days. This observation suggests that POCs could be candidates for housing artificial molecular machines with flexible extended limbs and mechanical stability under harsh conditions. The second one^271^ is the formation of dimeric and trimeric catenated cage cubes based on the weak interactions of the substituents (methoxy or thiomethyl groups) of the constituent 1,4-disubstituted terephthaldehydes in combination with solvophobic effects. These interlocked cage structures are very promising for future molecular switches or machines. In addition, Yuan and co-workers^272^ have engineered a “smart” catalytic system by locking catalytically active metal clusters within discrete cationic POCs, in which the counteranions provide the external stimuli before being passed along to trapped metal clusters. By employing external light and pH stimuli, this system shows excellent programmable activity control in various liquid-phase catalytic reactions. This result could inspire us to construct “smart” molecular machines for various chemical syntheses.

## 6 Outlook and Future Perspectives

The design and development of POCs with unique architectures and rich functionalities has become a rewarding new direction in the fields of chemistry, materials science, nanoscience, and nanotechnology. The recent surge in their development has benefited from breakthroughs in advanced analytical techniques, particularly in gas-adsorption and X-ray diffraction techniques. In this regard, there are already a few published reviews.^12,13,21,23,24,26,42,273−276^ Some of these reviews have focused on special types of POCs, such as multiporphyrinic^42^ or imine^274,275^ cages, while others have summarized the advent of new porous organic materials,^12,13,26^ involving POCs only in part. Others have covered one special aspect—for example, unique synthetic approaches,^21−23,276^ narrow functionalization angles,^24^ or small applications scope.^273^ In this review, we have described in detail the recent discoveries surrounding POCs as a result of their strategic design, precise synthesis, advanced characterization, and innovative applications. We believe that this relatively new class of porous materials can rival MOFs, COFs, and POPs in porosity and functionality. In addition, POCs offer new opportunities for applications in (mixed) films/membranes, porous liquids, and the homogenization of heterogeneous catalysts, mainly because of their outstanding solution processability. The field, however, is still immature and faces considerable challenges going forward. One stumbling block is the lack of a universal synthetic protocol. Irreversible linking chemistry produces robust POCs in quite low yields under kinetic control, while dynamic covalent chemistry provides high-yielding POCs but with low chemical and thermal stability since they are thermodynamically controlled products. This dichotomy constitutes a serious flaw since many industrial applications require both stability and scalability, with reactions taking place under harsh conditions—such as high temperature, pressure, acidity or alkalinity—while at the same time requiring kilogram-levels of materials at a minimum. It follows that it is highly desirable to develop versatile, high-yielding synthetic approaches for the construction of robust POCs.

Metal-free POCs, endowed with large pores and cavities in addition to unique functional backbones, can provide high-quality single crystals, which can be applied as excellent platforms in photoluminescence, drug delivery, photocatalytic and electronic applications. The one-pot, controllable synthesis of large single-crystal POCs, however, is still challenging. To add insult to injury, the key principles for POC cyclization and the understanding of structure–property relationships have not yet been elucidated. One approach could be to construct large single POC crystals by using standard, well-developed methods of organic crystal growth. Another approach could be to monitor closely the steps of cyclization and pore generation by using state-of-the-art optics, *in situ*, operando, or other innovative observation techniques. In addition, the retention of porosity of POCs after desolvation remains unresolved. While some molecular cages are robust after desolvation, most of them are much more delicate, and techniques such as supercritical drying or careful solvent exchange are required. New, facile, and general approaches are highly anticipated for solving such problems.

Computational chemistry is a facile and essential tool in modern science for predicting and designing POCs with particular functions. Unlike in the case of extended porous frameworks, computational investigations for POCs are rarely reported. One reason is that single-crystal structure prediction requires high-level calculations, which are not affordable to all researchers. Another reason is that crystal structure prediction of POCs remains a challenge for the future, particularly for cages that are not rigid. In this regard, POC-based systems are different than MOFs and COFs since there is no generalizable “isoreticular” strategy. It is both an advantage and a challenge—e.g., the interconverting polymorphs for switchable membranes^214^ makes de novo design a little more challenging. More reliable motifs are needed for supramolecular crystal design. One possible strategy is to place directing groups on the cage periphery such as in hydrogen-bonded organic frameworks (HOFs). New theoretical algorithms, simulations, theories, and methods are highly desirable when it comes to calculating complex guest–host interactions and bond-formation kinetics associated with POCs.

Although the applications of POCs in porous liquids and heterogeneous catalysis are still in their infancy, interest is set to rise going forward. In addition, the pore mechanism and synergistic interactions of cage and metal nanoparticles are not yet well established. Advanced visual observations will prove vital in the pursuit of these applications. In the meantime, the scope of POCs can be expanded based on new insights for understanding their solution behavior and processability.

In conclusion, thanks to the unwavering efforts of scientists of different persuasions, POCs have made significant inroads into finding uses. Sustainable research in this new area is clearly on the horizon with unique applications in industry, energy, and the environment looming large.
